# Imidazo[1,2-*b*]pyrazole-7-carboxamides Induce Apoptosis in Human Leukemia Cells at Nanomolar Concentrations

**DOI:** 10.3390/molecules23112845

**Published:** 2018-11-01

**Authors:** Gábor J. Szebeni, József A. Balog, András Demjén, Róbert Alföldi, Vanessza L. Végi, Liliána Z. Fehér, Imola Mán, Edit Kotogány, Barbara Gubán, Péter Batár, László Hackler, Iván Kanizsai, László G. Puskás

**Affiliations:** 1 Laboratory of Functional Genomics, Biological Research Centre, Hungarian Academy of Sciences, Temesvári krt. 62, H-6726 Szeged, Hungary; g.szebeni@avidinbiotech.com (G.J.S.); jozsef.a.balog@gmail.com (J.A.B.); vegi.vanessza@gmai.com (V.L.V.); kotogany.edit@brc.mta.hu (E.K.); 2 Department of Physiology, Anatomy and Neuroscience, Faculty of Science and Informatics, University of Szeged, Közép fasor 52, H-6726 Szeged, Hungary; 3 Avidin Ltd., Alsó kikötő sor 11/D, H-6726 Szeged, Hungary; a.demjen@avidinbiotech.com (A.D.); r.alfoldi@avidinbiotech.com (R.A.); l.feher@avidinbiotech.com (L.Z.F.); i.noktaman@avidinbiotech.com (I.M.); hackler@avidinbiotech.com (L.H.J.); i.kanizsai@avidinbiotech.com (I.K.); 4 Department of Dermatology and Allergology, University of Szeged, Korányi fasor 6, H-6720 Szeged, Hungary; gubanbarbi@gmail.com; 5 Department of Hematology, Institute of Internal Medicine, University of Debrecen, Nagyerdei Körút 98, 4032 Debrecen, Hungary; pbatar@med.unideb.hu

**Keywords:** imidazole, pyrazole, acute myeloid leukemia, apoptosis, toxicogenomics

## Abstract

Leukemia, the malignancy of the hematopoietic system accounts for 10% of cancer cases with poor overall survival rate in adults; therefore, there is a high unmet medical need for the development of novel therapeutics. Eight imidazo[1,2-*b*]pyrazole-7-carboxamides have been tested for cytotoxic activity against five leukemia cell lines: Acute promyelocytic leukemia (HL-60), acute monocytic leukemia (THP-1), acute T-lymphoblastic leukemia (MOLT-4), biphenotypic B myelomonocytic leukemia (MV-4-11), and erythroleukemia (K-562) cells in vitro. Imidazo[1,2-*b*]pyrazole-7-carboxamides hampered the viability of all five leukemia cell lines with different potential. Optimization through structure activity relationship resulted in the following IC_50_ values for the most effective lead compound DU385: 16.54 nM, 27.24 nM, and 32.25 nM on HL-60, MOLT-4, MV-4-11 cells, respectively. Human primary fibroblasts were much less sensitive in the applied concentration range. Both monolayer or spheroid cultures of murine 4T1 and human MCF7 breast cancer cells were less sensitive to treatment with 1.5–10.8 μM IC_50_ values. Flow cytometry confirmed the absence of necrosis and revealed 60% late apoptotic population for MV-4-11, and 50% early apoptotic population for HL-60. MOLT-4 cells showed only about 30% of total apoptotic population. Toxicogenomic study of DU385 on the most sensitive MV-4-11 cells revealed altered expression of sixteen genes as early (6 h), midterm (12 h), and late response (24 h) genes upon treatment. Changes in *ALOX5AP*, *TXN*, and *SOD1* expression suggested that DU385 causes oxidative stress, which was confirmed by depletion of cellular glutathione and mitochondrial membrane depolarization induction. Imidazo[1,2-*b*]pyrazole-7-carboxamides reported herein induced apoptosis in human leukemia cells at nanomolar concentrations.

## 1. Introduction

Leukemias and lymphomas accounts for almost 10% of cancer cases worldwide [1]. Leukemias are heterogeneous diseases due to differential cellular origin along with self-characteristic genetic abnormalities [2]. The discussion of current therapies is beyond the scope of the present article. Although bone marrow transplantation reached a break-through in the last decades for the treatment of childhood leukemias, chemotherapy remains the main option for adults, and especially for the elderly with much less therapeutic success nowadays. Based on the maturation status, differentiation level, and/or lineage commitment acute myeloid leukemia cells (AML) have been classified by the French-American-British classification (FAB) system [3,4,5]. To determine the specificity of imidazo[1,2-*b*]pyrazole-7-carboxamides as potential anti-leukemic agents we have focused on the following differentially matured AML cells: HL-60 acute promyelocytic leukemia, (FAB M2) [6,7], MV-4-11 biphenotypic B myelomonocytic leukemia (FAB M4) [8,9], THP-1 acute monocytic leukemia (FAB M5) [10,11], K-562 erythroleukemia, (FAB M6) [12,13]. One acute lymphoblastic leukemia cell line (ALL), the MOLT-4 acute T-lymphoblastic leukemia, was also involved in the study.

Pyrazole derivatives are pharmacologically relevant active scaffolds in diverse therapeutic fields, reviewed recently by Karrouchi et al. [14]. Moreover, the synthesis and the anticancer activity of *N*-fused pyrazoles, such as imidazo[1,2-*b*]pyrazoles have also been reported [15,16]. We have recently published the synthesis of a library (67 compounds) of novel imidazo[1,2-*b*]pyrazole-7-carboxamides, where among different cell lines, HL-60 leukemia cells showed the highest sensitivity upon treatment [17]. Based on the previous structure–activity relationship (SAR) experience, hit molecules have been further optimized by changing certain substituents to increase the cytotoxic effect against different leukemia cells. These efforts resulted in more active compounds with anti-leukemic potential reported first in this study.

## 2. Results

### 2.1. Synthesis of Imidazo[1,2-b]pyrazole-7-carboxamides

**Figure 1:**
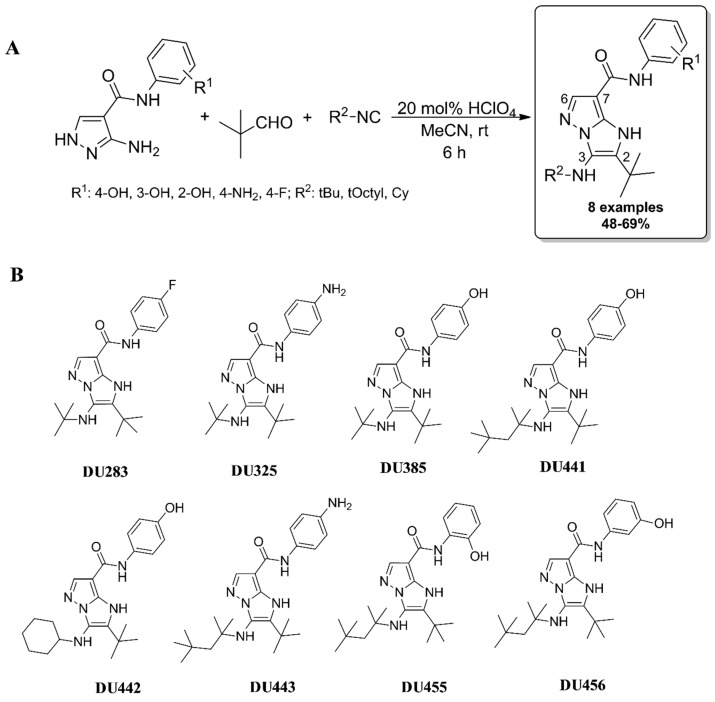
(**A**) General procedure for the preparation of imidazo[1,2-*b*]pyrazoles via Groebke-Blackburn-Bienaymé three components reaction (GBB-3CR). (**B**) Structures of imidazo[1,2-*b*]pyrazole-7-carboxamides.

Synthesis was performed by the previously presented manner via Groebke-Blackburn-Bienaymé three component reaction (GBB-3CR) [17]. Briefly, to a suspension of pyrazole precursor (0.50 mmol) in MeCN (0.5 mL), pivaldehyde (0.55 mmol), HClO_4_ (20 mol%), and the corresponding isocyanide (0.55 mmol) were added and stirred at room temperature for 6 h. Then the crude mixture was purified by filtration followed by washing with cold MeCN or by column chromatography on silica gel (eluent: hexane/EtOAc) to afford pure imidazo[1,2-*b*]pyrazoles (Figure 1A). Seven DU-compounds were prepared for antitumor characterization with a substitution pattern of C2 *tert*-butyl (*t*Bu), C3 aliphatic (*t*Bu, *tert*-octyl (*t*Octyl) or cyclohexyl (Cy)), and C7 phenyl substituted secondary carboxamides with electron-donating (ED) groups, such as hydroxyl (OH) and amino (NH_2_) functions (positioning in *ortho*-, *meta*- or *para* positions) (Figure 1B). In addition, one imidazo[1,2-*b*]pyrazole (DU283) incorporating electron-withdrawing (EWG) function in para position (R^1^ = 4-F) was selected among the investigated bicycles as an anti-tumor reference imidazo[1,2-*b*]pyrazole-7-carboxamide [17]. Spectral analysis of imidazo[1,2-*b*]pyrazole-7-carboxamides can be found in Figure S1.

### 2.2. Imidazo[1,2-b]pyrazole-7-carboxamides Hampered the Viability of Leukemia Cells with Different Potential

The substitutions chosen were based on the previously established structure–activity relationship (SAR) results [17], with the goal to design compounds with improved anti-leukemic effect. Seven compounds out of eight hampered the viability of all five tested leukemia cell lines with different potential. Superior efficiency was obtained for DU325 and DU385 (Table 1). The presence of *t*Bu as a bulky group in the R^2^ position in combination with 4-NH_2_ and 4-OH moieties in the R^1^ determined the strength of anti-leukemic efficiency. In addition, the hydroxyl moiety located in para position on the aromatic ring (R^1^ = 4-OH) provided the highest cytotoxicity (DU385). Optimization resulted in the following IC_50_ values for the most effective lead compound DU385: 16.54 nM, 27.24 nM, and 32.25 nM on HL-60, MOLT-4, MV-4-11, respectively (Table 1). 

In the case of DU441, incorporating a *t*Octyl function in R^2^ position, demonstrated an excellent anti-leukemic activity (60.48 nM IC_50_ value for MOLT-4). However, the tested DU443 with the same R^2^ substituent did not show a similar result (104.2 nM IC_50_ value for MOLT-4) (Table 1). Accordingly, the substitution of R^1^ = OH to NH_2_ (if R^2^ = tert-Octyl) decreased the measured biological effects (DU443). Incorporating an EWG group in the framework (R^1^ = 4-F; DU283) led to less active antitumor behavior (146.2 nM IC_50_ value for MOLT-4) (Table 1).

On the other hand, all 4-OH-phenyl substituted carboxamides proved to be very efficient against leukemia cell lines independent from the quality of R^2^ group. Each aliphatic functionalized, *t*Bu, *t*Octyl besides Cy (DU442) compounds with R^1^ = 4-OH demonstrated high cytotoxic potential (IC_50_ values were: 58.71 nM, 51.03 nM, 68.08nM on HL-60, MOLT-4, MV-4-11 cells, respectively (Table 1). Compounds (DU325, DU385, DU441, and DU442) with an IC_50_ value under 100 nM were selected for subsequent experiments (Table 1).

Interestingly, the anti-leukemic behavior was diminished in those where a hydroxyl group was in ortho or meta position (DU455 and DU456). The effect on human primary fibroblasts used as control cells were much less cytotoxic reducing the viability maximum to 60%, suggesting that the compounds reported herein were not as much as cytotoxic to non-malignant primary cells compared to leukemia cells (Table 1, Figure S7).

Cells were treated with imidazo[1,2-*b*]pyrazole-7-carboxamides in different concentrations (12.3 nM, 37 nM, 111 nM, 333 nM, 1 μM, 3 μM) in duplicates for 72 h. Viability was examined by a resazurin assay, as described in Section 4.4. Materials and Methods. Hashmark (#) labeled compounds were selected for further investigation. Dose response curves can be found in Figure S2 (HL-60), Figure S3 (MOLT-4), Figure S4 (MV-4-11), Figure S5 (THP-1), Figure S6 (K-562), Figure S7 (human primary fibroblasts). n.d. = not determined because the dose-response curves had a plateau around 0.7–0.6 (70–60%) viability with maximum 30–40% effect.

Cytotoxicity was further investigated on 4T1 murine mammary carcinoma and MCF7 human breast adenocarcinoma cancer cells to determine whether the cytotoxic effect was specific against leukemia cells or the compounds possessed a general antitumor effect. The four selected compounds diminished the viability of both 4T1 and MCF7 cells in micromolar concentrations irrespective of the culture method (minimum IC_50_ = 1.559 μM of DU442 on MCF7 2D, maximum IC_50_ = 10.8 μM of DU325 on 4T1 3D (Table 2). The micromolar effective dose is not pharmacologically feasible compared to the nanomolar activity against leukemias, so imidazo[1,2-*b*]pyrazole-7-carboxamides were considered as potential anti-leukemic drug candidates instead of general antitumoral compounds.

Cells were treated with imidazo[1,2-*b*]pyrazole-7-carboxamides in different concentrations (0.625 μM, 1.125 μM, 2.50 μM, 5 μM, 10 μM, 20 μM) in duplicates for 72 h. Viability was examined by resazurin assay as described in Section 4.4. Materials and Methods. Dose response curves can be found in Figure S8.

### 2.3. Leukemia Cells Died by Apoptosis Upon Treatment by Imidazo[1,2-b]pyrazole-carboxamides

**Figure 2:**
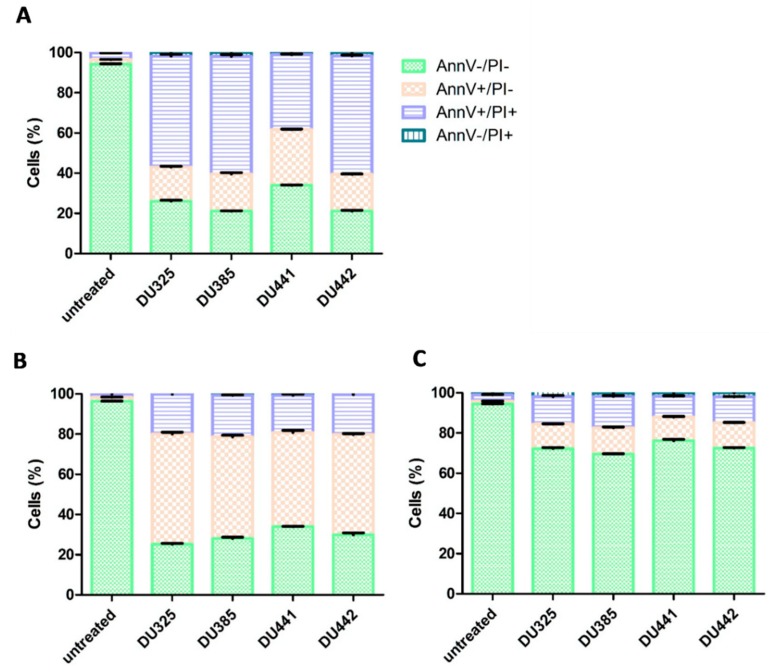
Imidazo[1,2-*b*]pyrazole-7-carboxamides induced phosphatidylserine exposure on human leukemia cells after 24 h. (**A**) MV-4-11, (**B**) HL-60, (**C**) MOLT-4 cells were incubated with 200 nM imidazo[1,2-*b*]pyrazole-7-carboxamides as described in Section 4.5 Materials and Methods. The results are shown as arithmetic mean values of the summary of living cells (AnnV−/PI−), early (AnnV+/PI−) and late apoptotic cells (AnnV+/PI+) of three samples ± SD. Necrosis (AnnV−/PI+) did not occur. Corresponding flow cytometry data (SSC-FSC and AnnV/propidium iodide dot plots) can be found in Figure S9 (MV-4-11), Figure S10 (HL60), Figure S11 (MOLT-4).

To determine whether the anti-leukemic effect of imidazo[1,2-*b*]pyrazole-7-carboxamides relies on apoptosis or necrosis flow cytometry Annexin V propidium iodide staining was performed. Four compounds with IC_50_ values under 100 nM have been selected for apoptotic studies on the most sensitive three cell lines (HL-60, MOLT-4, MV-4-11). Flow cytometry confirmed the absence of necrosis and revealed about 55%, 60%, 40%, 60% late apoptotic population (AnnV+/PI+) for MV-4-11 cells upon treatment by DU325, DU385, DU441, DU442, respectively (Figure 2A). While all compounds induced only 20% late apoptotic population (AnnV+/PI+) for HL-60 cells, the early apoptotic populations (AnnV+/PI−) for HL-60 cells were about 55%, 50%, 45%, 50% upon treatment by DU325, DU385, DU441, DU442, respectively (Figure 2B). This suggests that MV-4-11 cells are the most sensitive to drug candidates. MOLT-4 cells showed only about 25–30% of total apoptotic population (early + late) indicating that lymphoid leukemias are less sensitive toward apoptosis than myeloid leukemia cells (Figure 1C). 

### 2.4. Toxicogenomic Data upon Treatment by Imidazo[1,2-b]pyrazole-7-carboxamide DU385

To have a deep insight into the disturbance of cellular homeostasis of leukemic cells due to treatment by imidazo[1,2-*b*]pyrazole-7-carboxamides we have designed a toxicogenomic panel for a gene expression study (Table S1). The most sensitive MV-4-11 cells were treated by the most active lead compound DU385 as described in the Section 4.6 Materials and Methods. Functional categories of the investigated genes have been listed in Table S2. The toxicogenomic panel consists of genes which have been previously reported by Puskas et al. [18] and Zhang et al. in connection with drug induced cytotoxicity: Among others *EGR1*, *GDF15*, *ATF3*, *FGF21* [19]. The expression of genes, published by Wang et al. involved in the differentiation and cytotoxicity of HL-60 cells induced by retinoids was also monitored in our study: *SLC21A3*, *LGALS1*, *LBR*, *ALOX5AP*, *TXN*, *UBC*, *BCL2A1*, *CALR* [20]. We implemented genes from our previous toxicogenomic study: *ANXA2*, *GADD153*, *HSPA1A*, *SOD1* [21]. G-protein coupled receptor 84 (*GPR84*), a key player of β-catenin mediated signaling maintaining leukemogenesis was also investigated [22]. Finally, the expression of interleukin-6 (*IL6*) and tumor necrosis factor alpha (*TNF*) inflammatory cytokines was investigated [23,24].

**Figure 3:**
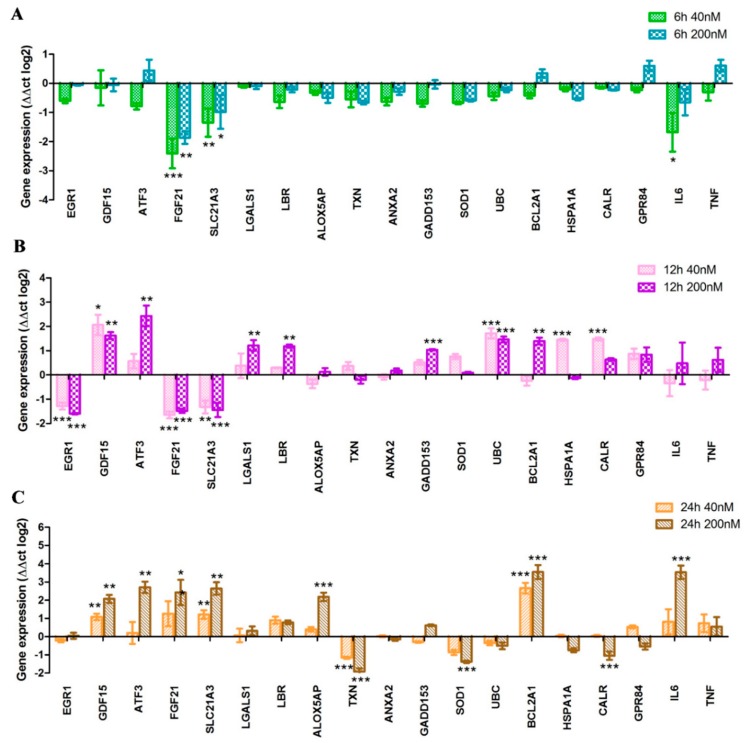
The expression of genes associated with drug induced cytotoxicity has been studied by quantitative real-time PCR after treatment with 40 nM or 200 nM DU385 compound for 6 h (**A**), 12 h (**B**), and 24 h (**C**), as described in Section 4.6 Materials and Methods. * *p* < 0.05, ** *p* < 0.01, *** *p* < 0.001.

The determined temporal pattern of differential expression divided the tested genes into three groups as: Early (6 h) (Figure 3A), midterm (12 h) (Figure 3B), and late response (24 h) genes upon treatment (Figure 3C).

As an early stress response, fibroblast growth factor 21 (*FGF21*), solute carrier organic anion transporter 1A2 (*SLC21A3*), and *IL6* genes were repressed 5.6 (log_2_ = −2.5), 3.4 (log_2_ = −1.8), and 2.25 (log_2_ = −1.5) times upon 40 nM treatment, respectively (Figure 3A). After 12 h incubation, not only *FGF21* and *SLC21A3*, but also early growth response 1 (*EGR1*), showed decreased expression (2.8 times repression, log_2_ = −1.5) (Figure 3B). It proved the concept that imidazo[1,2-*b*]pyrazole-7-carboxamides perturb the homeostasis of leukemia cells; nine genes (*GDF15*, *ATF3*, *LGALS1*, *LBR*, *GADD153*, *UBC*, *BCL2A1*, *HSPA1A*, *CALR*) associated with drug induced cytotoxicity have been significantly upregulated after 12 h stimulation (Figure 3B). Among the late response genes, some associated with oxidative stress were dysregulated, *ALOX5AP* increased 4 times (log_2_ = 2), whilst *TXN* and *SOD1* downregulated around 4 times (log_2_ = −2 and log2 = −1.8, respectively) upon 200 nM 24 h treatment (Figure 3C). The expression of the endoplasmic reticulum associated chaperon, calreticulin (*CALR*), also decreased (log_2_ = −1.1). Probably as a compensatory mechanism, the expression of *FGF21* (log_2_ = 2.4), *SLC21A3* (log_2_ = 2.6) and *BCL2A1* elevated (log2 = 3.5). Interleukin-6 (*IL6*) has been recently reported to contribute to the chemoresistance of pediatric AML [23], and we showed the overexpression (log_2_ = 3.3) of *IL6* after 24 h treatment (Figure 3C).

### 2.5. Imidazo[1,2-b]pyrazole-7-carboxamide DU385 Exerted Oxidative Stress of MV-4-11 Cells

**Figure 4:**
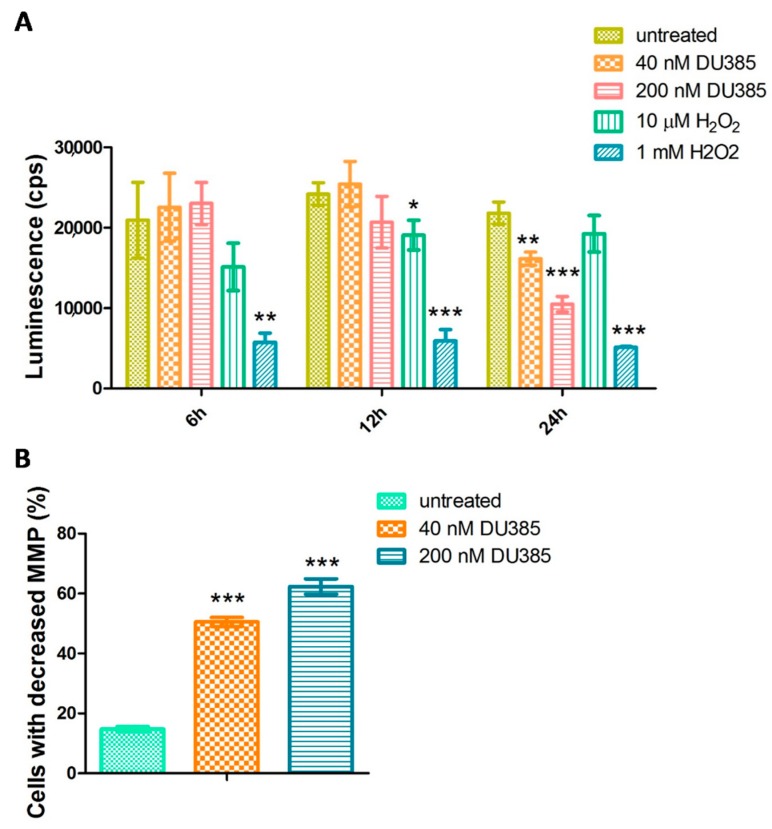
Imidazo[1,2-*b*]pyrazole-7-carboxamide DU385 exerted oxidative stress of MV-4-11 cells. Reduction of the glutathion (GSH) level (**A**) and the loss of the mitochondrial membrane potential (MMP) (**B**) were detected after 24 h as described in Section 4.7 Materials and Methods. * *p* < 0.05, ** *p* < 0.01, *** *p* < 0.001.

Changes in the gene expression of oxidative stress-related genes (*ALOX5AP*, *TXN*, *SOD1*) opened the way for the investigation of cellular glutathione (GSH) level upon treatment. The luminescence in the assay is proportional with the GSH level, which can be reduced by reactive oxygen species (ROS) via oxidation or reaction with the thiol group [25]. Hydrogen peroxide validating the assay as a positive control was effective to decrease the GSH level prominently in 1 mM after 6, 12, and 24 h (Figure 4A). The imidazo[1,2-*b*]pyrazole-7-carboxamide, DU385, was effective to generate ROS and the loss of GSH after 24 h to the 75% or 50% of the untreated cells with 40 nM and 200 nM treatment, respectively. 

Since oxidative stress may subsequently confound mitochondrial homeostasis, the mitochondrial membrane potential was measured by JC-1 assay. After 24 h, the percentage of cells with decreased mitochondrial membrane potential three and four times increased after the treatment with 40 nM or 200 nM DU385, respectively (Figure 4B, Figure S12). 

## 3. Discussion

We have shown the anti-leukemic effect of imidazo[1,2-*b*]pyrazole-7-carboxamides (Figure 1 and Figure S1) using the resazurin viability assay (Table 1, Figures S2–S6). The dose-response curves of the lead compound DU385 determined the following IC_50_ values: 16.54 nM, 27.24 nM, and 32.25 nM on HL-60, MOLT-4, MV-4-11 cells, respectively. Importantly, human primary fibroblasts were much less sensitive in the applied concentration range (12.3 nM–3 μM) suggesting selective cytotoxic effect, especially on leukemia cells (Table 1, Figure S7). Since carcinoma cells establish solid tumors in vivo, breast cancer cells were grown as three-dimensional (3D) spheroids compared to traditional tissue culture dishes (2D) (Table 2, Figure S8). Monolayer cultures ignore the role of microenvironmental factors, such as structural niche, with the plethora of molecular and cellular constituents [26]. Three dimensional cell cultures as drug discovery models better represent the in vivo situation like the partial oxygen tension, local pH, or extracellular matrix composition [27]. Although monolayer cultures were much sensitive to treatment, the effect was much lower (between 1.5–10.8 μM IC_50_ values, Table 2) both in 2D and 3D models of murine 4T1 or human MCF7 breast carcinoma cells, compared to the nanomolar IC_50_ values in leukemias (Table 1).

Incorporating R^1^ hydroxyl group in para position with a *t*Bu (DU385), *t*Octyl (DU441), or Cy (DU442) functions in R^2^ position, demonstrated an excellent anti-leukemic activity. Moreover, R^1^ para NH_2_ group combined with R_2_ *t*Octyl (DU325) had an anti-leukemic effect at nanomolar concentration. This potent anti-leukemic activity was diminished in those compounds where the R^1^ hydroxyl group was in ortho or meta position (DU455 and DU456), or the R^2^ *t*Octyl function was combined with the R^1^ NH_2_ group (DU443). Incorporating an EWG group in the framework (R^1^ = 4-F; DU283) led to a less active analog. All four selected compounds, DU325, DU385, DU441, and DU442 induced apoptosis of biphenotypic B myelomonocytic leukemia MV-4-11, acute promyelocytic leukemia HL-60, and the T-lymphoblastic leukemia MOLT-4 cells with different potential (Figure 2 and Figures S9–S11). None of the compounds induced necrosis. Myeloid cells were more sensitive to imidazo[1,2-*b*]pyrazole-7-carboxamides than the lymphoid MOLT-4, regarding the apoptotic process detected by flow cytometry.

Toxicogenomic screen was performed on MV-4-11 cells, the most susceptible cells for apoptosis. MV-4-11 cells were treated by the lead compound DU385. We have determined the expression pattern of early (6 h), midterm (12 h), and late response (24 h) genes upon imidazo[1,2-*b*]pyrazole-7-carboxamide stress (Figure 3). Early stress-related genes, FGF21 and SLC21A3 were repressed at both 6 h and 12 h treatments complemented with the decline of EGR1 expression at 12 h (Figure 3A,B). Early endoplasmic reticulum stress-associated genes, activating transcription factor 3 (ATF3), and DNA damage inducible transcript 3 (GADD153), elevated with chaperons like calreticulin (CALR) and heat shock protein family A (Hsp70) member 1A (HSPA1A) from 12 h of incubation. Other genes associated with differentiation and cell adhesion: Growth differentiation factor 15 (GDF15), galectin-1 (LGALS1), lamin B receptor (LBR) were upregulated at 12 h. Polyubiquitin genes and Bcl family members regulate cell survival and apoptosis [28,29], ubiquitin C has been described as a stress-response gene [30,31] activated at 12 h in MV-4-11 cells. The apoptosis regulator BCL2A1 (Bfl-1/A1) also elevated at 12 and 24 h upon stimulation, which has been described as anti-apoptotic [32], but also as pro-apoptotic via proteasome mediated turnover or calpain like cleavage [33]. During apoptosis detected at 24 h, genes responsible for oxidative stress response were dysregulated, ALOX5AP increased, and TXN and SOD1 downregulated upon 200 nM 24 h treatment (Figure 3C). Elevated levels of GDF15, ATF3, FGF21, SLC21A3, and IL6, were coupled with 20% early and 60% late apoptotic cells at 24 h (Figure 2A).

Changes in the gene expression of oxidative stress-related genes (ALOX5AP, TXN, SOD1) initiated the investigation of cellular GSH level. The amount of intracellular GSH dropped upon DU385 treatment only after 24 h. The consistency in the GSH level with DU385 treatment after 6 and 12 h may indicate that the generation of ROS is not a fast stress response (like to H_2_O_2_), rather a well-organized process after 24 h. Since oxidative stress induces mitochondrial dysfunction, and disturbance in the mitochondrial membrane potential can lead to apoptosis [34], the MMP was measured upon treatment by JC-1 assay. We have shown that mitochondria of MV-4-11 leukemia cells were depolarized when treated with 40 nM or 200 nM DU385 after 24 h.

Since leukemia cells used in this study were immature cells, malignant cells of myeloid or lymphoid precursors, authors may speculate that cells differentiate after treatment and the mitochondrial pathway of apoptosis is followed after differentiation (Figure 4). Granulation (side scatter = SSC) of the treated MV-4-11 (Figures S9 and S12), HL-60 (Figure S10) and MOLT-4 (Figure S11) cells increased after treatment, which proposes the differentiation concept. Apoptosis followed after differentiation to chemotherapy has already been published for immature leukemias [35,36,37], e.g., all-trans retinoic acid (ATRA) acts via that mechanism, which is applied in the clinical protocols [38]. Authors hypothesize that human fibroblasts and mouse 4T1 or human MCF7 breast carcinoma cells are less sensitive to DU385, probably because these are already differentiated cells with less apoptotic susceptibility or more complex anti-apoptotic mechanisms. Anyhow, further research is needed to reveal the cause of the sensitivity of immature leukemias to imidazo[1,2-*b*]pyrazole-7-carboxamide DU385.

In conclusion, imidazo[1,2-*b*]pyrazole-7-carboxamides reported herein, causing oxidative stress and apoptosis in nanomolar concentrations are promising drug candidates against human myeloid leukemias.

## 4. Materials and Methods

### 4.1. Ethical Statement 

Participant informed consent was obtained prior to surgical intervention for the isolation of human primary fibroblasts. All tissue collection complied with the Guidelines of the Helsinki Declaration and was approved by the Regional and Institutional Research Ethics Committee (2799, 3517).

### 4.2. Skin Biopsies and Cell Culture of Human Primary Fibroblasts

Healthy volunteers (age 18–60 years) were enrolled into the study. The punch biopsies were taken from healthy subjects from the breast area undergoing plastic surgery. Primary fibroblasts were obtained from the skin by enzymatic digestion according to a standard protocol. Briefly, skin specimens were first washed in Salsol A solution (Human Rt, Gödöllő, Hungary) supplemented with 2% antibiotic/antimycotic solution (Sigma-Aldrich, St. Louis, MO, USA). Skin samples were then cut into narrow strips and incubated in Dispase solution (Roche Diagnostics, Mannheim, Germany) overnight at 4 °C. The epidermis was subsequently separated from the dermis. Fibroblasts were obtained by incubating the dermis in Digestion Mix solution (Collagenase, Hyaluronidase and Deoxyribonuclease) for 2 h at 37 °C. Cell suspensions were filtered through a 100 µm nylon mesh (BD Falcon, San Jose, CA, USA), and cells were pelleted by centrifugation (Thermo Fisher Scientific, Waltham, MA, USA, Megafuge 16). Fibroblasts were grown in low glucose DMEM/F12 medium containing 15% FCS, 1% antibiotic/antimycotic (PAA, Pasching, Austria) and 1% l-glutamine solution (PAA). Fibroblasts were cultured at 37 °C and 5% CO_2_ in humidified conditions. Depending on the cell growth, the medium was changed every 2–4 days, and cells were passaged at 80% of confluence. 

### 4.3. Cell Culturing, 3D Spheroid Formation and Treatments

The following cells were purchased from the American Type Culture Collection (ATCC, Manassas, WV, USA): HL-60 acute promyelocytic leukemia, THP-1 acute monocytic leukemia, MOLT-4 acute T-lymphoblastic leukemia, MV-4-11 biphenotypic B myelomonocytic leukemia, and K-562, erythroleukemia maintained in RPMI 10% FCS (Gibco, Thermo Fisher Scientific, Waltham, MA, USA) using tissue culture dishes (Corning Life Sciences, Corning, NY, USA). The human breast adenocarcinoma MCF-7 and mouse mammary carcinoma 4T1 cells were also purchased from the ATCC. The MCF-7 cells were maintained in Dulbecco’s Modified Eagle Medium/Nutrient Mixture F-12 (DMEM/F12) 10% fetal calf serum (FCS, Gibco), and the 4T1 were maintained in Roswell Park Memorial Institute 1640 medium (RPMI-1640) with 10% FCS. The pH of the cell culture media was controlled to be between 7.2–7.4 prior use. All used media were supplemented with 2 mM GlutaMAX, and 100 U/mL penicillin, 100 µg/mL streptomycin (Life Technologies, Carlsbad, CA, USA) before use. Cells were passed every three days and placed in a humidified incubator at 37 °C 5% CO_2_ (Sanyo, Osaka, Japan).

The 3D spheroid production using pellet culture system was previously described by Johnstone, B et al. [39]. Briefly, the MCF-7 and 4T1 cells were gently detached from the conventional tissue culture flasks using trypsin, washed with PBS, counted, and then suspended to have 6000 cells in 100 μL medium per well. The cellular suspensions were dispended into cell repellent, U shaped CellStar^®^ 96 microplates (Cellstar^®^ Cell-Repellent Microplate, Greiner Bio-One, Kermsmünster, Austria) and centrifuged at 1200 *g* for 10 min. After spheroid formation (one spheroid/well), the spherical aggregates were directly used for viability assay without any detaching procedures and transfer steps. The cell repellent plates were incubated and maintained together with conventional 2D cell culture plates at 37 °C in a humidified incubator in an atmosphere of 5% CO_2_ (Sanyo).

Compounds were dissolved in dimethyl sulfoxide (DMSO) at 10 mM concentration freshly before being used. Since DMSO can be toxic for cellular systems above 1%, the stock solution was further diluted in serial dilutions in all cases in the appropriate cell culture media. The intermediate dilution of compounds for Figure 1 was 15 µM (666.7× dilution) in the appropriate cell culture media, and it was three times serially diluted to 5 µM, 1.667 µM, 555 nM, 185 nM, 61.7 nM, then each intermediate dilution was further diluted 5× when it was added to the cells, so the treatments were 3 µM, 1 µM, 333 nM, 111 nM, 37.5 nM, 12.3 nM. The intermediate dilution of compounds for Figure 2, Figure 3 and Figure 4 was 100 µM (100× dilution), it was serially diluted to 1 µM (100× dilution), and it was further diluted to 200 nM (5× dilution), then each intermediate dilution was further diluted 5× when it was added to the cells, so the treatments were 200 nM (5×), 40 nM (5×).

### 4.4. Resazurin Viability Assay

The human primary fibroblasts, 4T1 or MCF7 cells (6000), and leukemia (HL-60, MOLT-4, MV-4-11, THP-1, K-562) cells (20,000) were seeded into 96-well plates (Corning Life Sciences, Corning, NY, USA) in media. Adherent cells were cultured overnight before treatment. Effects of DU compounds were examined in the following concentrations: 12.3 nM, 37 nM, 111 nM, 333 nM, 1 μM, 3 μM in 100 µl after 72 h incubation, for leukemias and control human primary fibroblasts. Treatment conditions were 0.625 μM, 1.125 μM, 2.50 μM, 5 μM, 10 μM, 20 μM for 4T1 or MCF7 cells, for 72 h. Viability assay was carried out as described previously in Reference [40]. Briefly, resazurin reagent (Sigma-Aldrich) was dissolved in PBS (pH 7.4) at 0.15 mg/mL concentration, sterile filtered (0.22 µm, Merck Millipore), and aliquoted at −20 °C. We applied resazurin 20 μL stock to 100 μL/well culture. After 2 h incubation at 37 °C, 5% CO_2_ (Sanyo) fluorescence (530nm excitation/580nm emission) was recorded on a multimode microplate reader (Cytofluor4000, PerSeptive Biosytems, Framingham, MA, USA). Viability was calculated with relation to untreated control cells (1 corresponds to 100% viability on the *y* axis, Figures S2–S8), and blank wells containing media without cells. IC_50_ values (50% inhibiting concentration) were calculated by GraphPad Prism^®^ (version 5.01, La Jolla, CA, USA).

### 4.5. Detection of Phosphatidylserine Exposure

Apoptosis was measured by flow cytometry as described previously in References [40,41]. Briefly, cells (200,000) were plated in 24-well tissue culture plates (Corning Life Sciences) and treated with the indicated compounds at 200 nM in 500 μL media. After 24 h, the supernatants were harvested. Cells were harvested with the corresponding supernatant and centrifuged down (2000 rpm, 5 min, Eppendorf, Hamburg, Germany). Pellet was resuspended in Annexin V binding buffer (0.01 M HEPES, 0.14 M NaCl and 2.5 mM CaCl_2_). Annexin V-Alexa Fluor^®^ 488 (Life Technologies, 2.5:100) was added to the cells, which were then kept for 15 min in the dark at room temperature. Before the acquisition, propidium iodide (10 μg/mL, Sigma-Aldrich, St. Louis, MO, USA) was added in Annexin V binding buffer to dilute Annexin V-Alexa Fluor^®^ 488 5×. Cells (20,000 events) were analyzed on a FACSCalibur flow cytometer using CellQuest software (version 3.3, Becton Dickinson, Franklin Lakes, NJ, USA). The percentage of the FL1 (530/30 nm filter, Annexin V-Alexa Fluor^®^ 488) positive, and FL3 (670 nm filter, propidium iodide) negative early apoptotic cells and FL1 positive and FL3 positive late apoptotic cells were determined. The total apoptotic population included both early and late apoptotic cells. Column charts were created by GraphPad Prism^®^ 5. Corresponding flow cyrometry data (SSC-FSC and AnnV/propidium iodide dot plots) can be found in Figure S9 (MV-4-11), Figure S10 (HL60), Figure S11 (MOLT-4).

### 4.6. Quantitative-Real Time PCR

MV-4-11 cells (10 × 10^6^) were plated in 100 mm tissue culture dishes (Corning Life Sciences, Corning, NY, USA) in RPMI 10% FCS. At different time points, cells were treated in 10 mL total volume with test compound DU385 at 40 nM or 200 nM for 6 h, 12 h, or 24 h. After treatment, nucleic acid preparation was done by using the RNA purification kit (Direct-zolTM RNA MiniPrep Kit, Zymo Research, Irvine, CA, USA), according to an already published protocol in Reference [40]. The quality and quantity of the isolated RNA were measured with NanoDrop1000 Version 3.8.1. (Thermo Fisher Scientific, Waltham, MA, USA). Reverse transcription from 3 µg of total RNA was performed with the High-Capacity cDNA Archive Kit (Applied Biosystems, Foster, CA, USA) in a total volume of 30 µL according to the manufacturer’s protocol. After dilution with 130 μL of ultrapure water (Applied Biosystems), cDNA was used as template for gene expression analysis. Quantitative real-time PCR (qRT-PCR) was performed on the LightCycler^®^ 96 System (Roche, Basel, Switzerland), using gene-specific primers with SYBR Green protocol, as described previously in Reference [42]. Briefly, for cycling, each 10 μL PCR reaction contained 1 µL cDNA (18.75 ng), 250 nM primers, and 5 μL qPCRBIO SyGreen Mix Lo-ROX (2×, PCR Byosystems, London, UK). Primer sequences and accession numbers are listed in Table S1. The PCR protocol was as follows: Enzyme activation at 95 °C for 2 min, 45 cycles of denaturation at 95 °C for 10 s, annealing at 60 °C, and extension at 60 °C for 10 s. All the PCRs were performed with three replicates. After amplification, the melting curve was checked to verify the specificity of the PCR reactions. The Ct values were normalized to GAPDH gene for each time point. The presented relative gene expression ratios were ΔΔCT values (log_2_). All values were presented as mean ± standard deviation (SD). 

### 4.7. Detection of the Oxidative Stress

Glutathion measurement was carried out as previously described in Reference [43]. MV-4-11 cells were plated (2 × 104) in a 96-well tissue culture plate (Corning Life Sciences, Corning, NY, USA), and were incubated with 40 nM, 200 nM DU385 compound or 10 μM and 1 mM H_2_O_2_ (Sigma-Aldrich) in RPMI 10% FCS for 6, 12, and 24 h, in a humidified atmosphere of 95% air and 5% CO_2_. Cells were harvested, washed in PBS, centrifuged (2000 rpm, 5 min), suspended in 100 μL PBS. Then, 50 μL aliquots of prepared 2× GSH-GloTM Reagent (GSH-GloTM Glutathione assay; Promega, Madison, WI, USA) were added to 50 μL of cells and incubated at room temperature in a black 96-well microtiter plate (Tomtec, Budapest, Hungary) for 30 min. Thereafter, 100 μL of reconstituted Luciferin Detection Reagent (Promega, Medison, WI, USA) was added to each well, and cells were incubated for 15 min further. The amount of light produced (cps = counts per second) was detected by a plate reader (1420 Victor, Wallac, Perkin Elmer, Waltham, MA, USA). Data were visualized by a GraphPad Prism^®^ 5.

Mitochondrial membrane potential was measured as described previously in Reference [44]. Briefly, MV-4-11 cells (2 × 10^5^) were plated in 24-well tissue culture plates (Corning Life Sciences, Corning, NY, USA) in RPMI 10% FCS and were treated in 500 μL media containing 40 nM or 200 nM DU385 compound. Untreated controls cells were supplemented with 500 μL cell culture media. After 24 h, the cells were harvested and centrifuged (2000 rpm, 5 min). The pellet was suspended and incubated for 15 min in 5 μg/mL JC-1 (5,5′,6,6′-tetrachloro-1,1′,3,3′-tetraethylbenzimidazolocarbocyanine iodide, Chemodex, St. Gallen, Switzerland) containing media in final volume 300 μL at 37 °C. Finally, using FL2 (cells with steady state mitochondria) (585/42 nm)–FL1 (cells with depolarized mitochondria) (530/30 nm) channels, the red–green fluorescence intensity of 2 × 10^4^ events was acquired immediately on a FACSCalibur flow cytometer. Data were analyzed using CellQuestTM software (CellQuest Pro v5.1, Becton Dickinson, Franklin Lakes, NJ, USA). Bar graphs showed the percentage of FL1 positive cells visualized by GraphPad Prism^®^ 5. 

### 4.8. Statistical Analysis

Statistical analysis was performed using two-tailed, heteroscedastic Student′s *t*-test to evaluate the statistical significance (set at * *p*< 0.05, ** *p* < 0.01, *** *p* < 0.001) between two given experimental groups: Pairwise comparison of each sample to the untreated control.

## Figures and Tables

**Table 1:** IC_50_ values (nM) of analogs determined by resazurin assay upon treatment by imidazo[1,2-*b*]pyrazole-7-carboxamides.

	HL-60	MOLT-4	MV-4-11	THP-1	K-562	Human Fibroblasts
**DU283**	266.9	146.2	209.4	352.5	493.7	n.d.
**DU325 ^#^**	66.31	39.35	50.21	73.78	194.9	n.d.
**DU385 ^#^**	16.54	27.24	32.25	25.88	54.31	n.d.
**DU441 ^#^**	62.04	60.48	87.56	94.9	190.9	n.d.
**DU442 ^#^**	58.71	51.03	68.08	95.22	163.6	n.d.
**DU443**	130.7	104.2	173.7	108.9	303.1	n.d.
**DU455**	2169	1211	2892	2354	inactive	n.d.
**DU456**	446.5	324.8	524.5	761.4	901.8	n.d.

**Table 2:** IC_50_ values (μM) of analogs on 4T1 and MCF7 breast cancer cells determined by resazurin assay upon treatment by imidazo[1,2-*b*]pyrazole-7-carboxamides.

	4T1 2D	4T1 3D	MCF7 2D	MCF7 3D
**DU325**	3.938	10.800	3.445	8.216
**DU385**	4.558	9.520	3.531	8.853
**DU441**	3.363	4.914	3.032	3.537
**DU442**	2.255	5.656	1.559	5.514

## Supplementary Materials

The following are available online, Figure S1: Spectral data of imidazo[1,2-*b*]pyrazole carboxamides, Figure S2: Dose-response curves of imidazo[1,2-*b*]pyrazole-7-carboxamides on HL-60 cells, Figure S3: Dose-response curves of imidazo[1,2-*b*]pyrazole-7-carboxamides on MOLT-4 cells, Figure S4: Dose-response curves of imidazo[1,2-*b*]pyrazole-7-carboxamides on MV-4-11 cells, Figure S5: Dose-response curves of imidazo[1,2-*b*]pyrazole-7-carboxamides on THP-1 cells, Figure S6: Dose-response curves of imidazo[1,2-*b*]pyrazole-7-carboxamides on K-562 cells, Figure S7: Dose-response curves of imidazo[1,2-*b*]pyrazole-7-carboxamides on human primary fibroblast cells, Figure S8: Dose-response curves of imidazo[1,2-*b*]pyrazole-7-carboxamides on murine (4T1) and human (MCF7) breast cancer cells grown as a monolayer (2D) or spheroid (3D), Figure S9: Detection of phosphatidylserine exposure on MV-4-11 cells, Figure S10: Detection of phosphatidylserine exposure on HL-60 cells, Figure S11: Detection of phosphatidylserine exposure on MOLT-4 cells, Figure S12: Detection of the mitochondrial membrane potential.
