# Supplementary material for: Imidazo[1,2-b]pyrazole-7-carboxamides Induce Apoptosis in Human Leukemia Cells at Nanomolar Concentrations
Source: Molecules. 2018 Nov 1;23(11):2845. doi: 10.3390/molecules23112845 (PMC6278434; doi:10.3390/molecules23112845)
Supplement: Supplementary file 1 [file molecules-23-02845-s001.zip › molecules-372705-SI/Supplement Revised/FigureS1.pdf]

Figure S1 Spectral data of imidazo[1,2-*b*]pyrazole carboxamides

2-(Tert-butyl)-3-(tert-butylamino)-N-(4-fluorophenyl)-1H-imidazo[1,2-b]pyrazole-7-carboxamide **(DU283)**

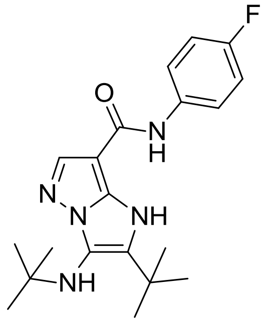

Gray solid; yield: 49%; m.p. 190 °C; ^{1}H NMR (500 MHz, DMSO-d_6) δ 11.08 (s, 1H), 9.50 (s, 1H), 8.11 (s, 1H), 7.73 (dd, *J* = 9.0, 5.0 Hz, 2H), 7.15 (t, *J* = 8.7 Hz, 2H), 3.69 (s, 1H), 1.42 (s, 9H), 1.22 (s, 9H). ^{13}C NMR (126 MHz, DMSO-d_6) δ 161.4, 158.1 (d, *J* = 238.9 Hz), 140.8, 137.4, 136.7, 133.2, 121.8 (d, *J* = 7.8 Hz), 120.3, 115.5 (d, *J* = 22.0 Hz), 94.2, 52.9, 32.5, 31.2, 30.7. ESI-MS (m/z): 372.2 (M+H$^+$).

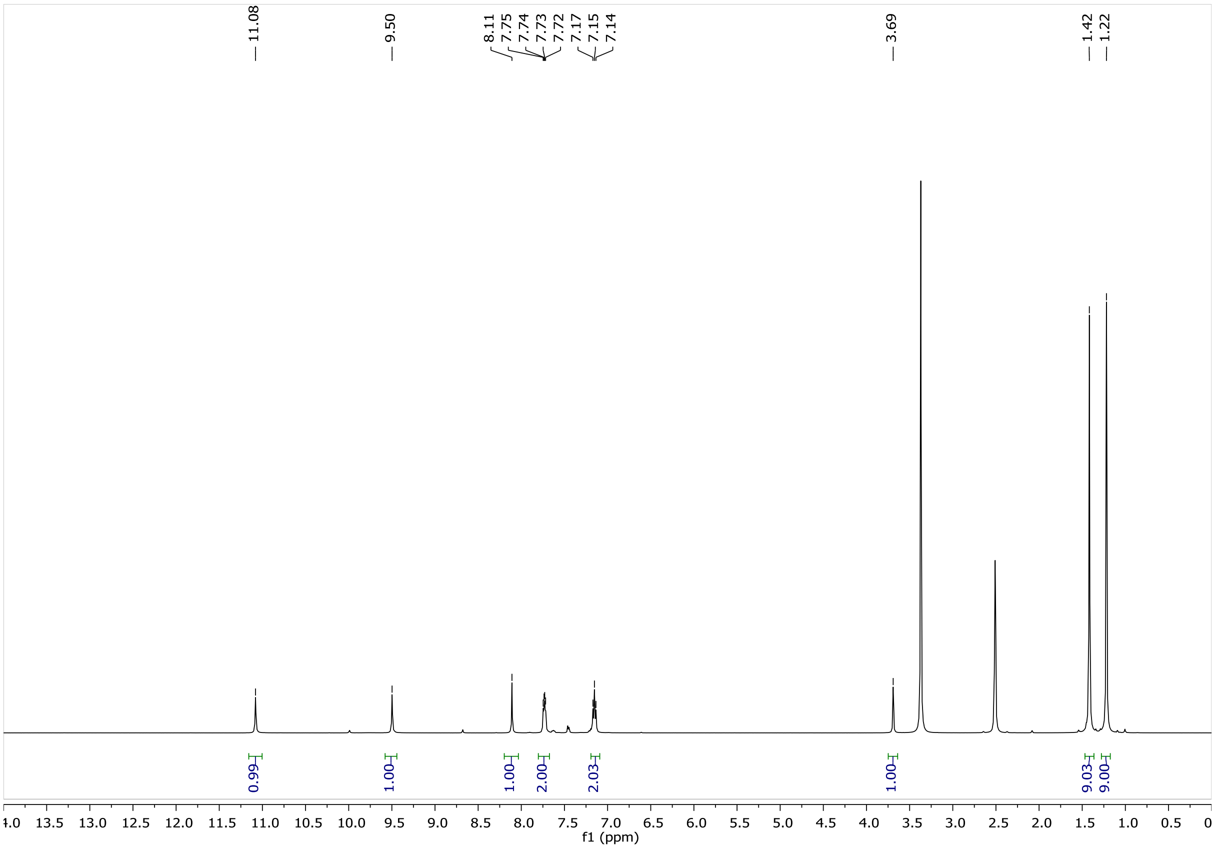

^{1}H NMR spectra of **DU283**

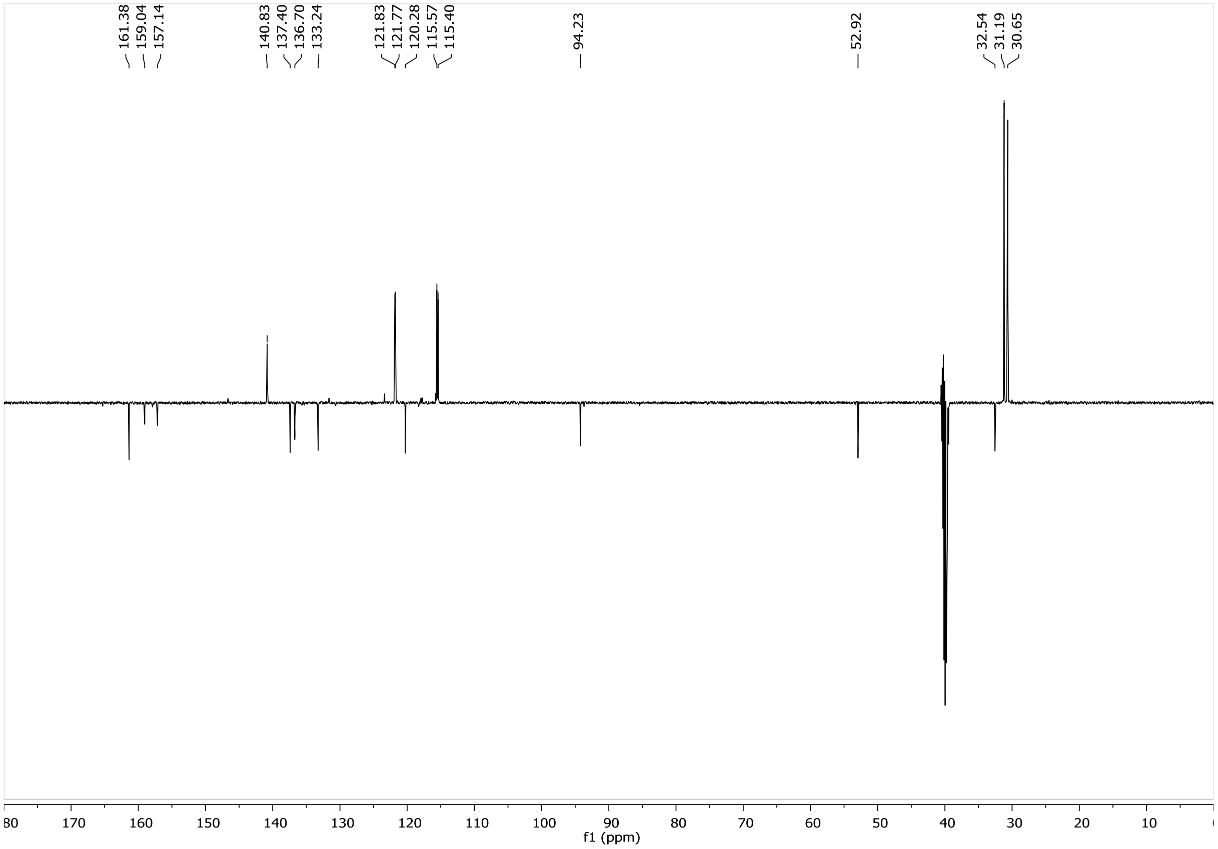

^{13}C NMR spectra of **DU283**

***N-(4-Aminophenyl)-2-(tert-butyl)-3-(tert-butylamino)-1H-imidazo[1,2-b]pyrazole-7-carboxamide* (DU325)**

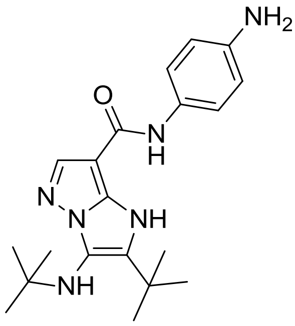

Gray solid; yield: 56%; m.p. 198 °C; ^{1}H NMR (500 MHz, DMSO-*d*$_6$) δ 10.93 (s, 1H), 9.04 (s, 1H), 8.02 (s, 1H), 7.29 (d, *J* = 8.6 Hz, 2H), 6.53 (d, *J* = 8.5 Hz, 2H), 4.83 (s, 2H), 3.66 (s, 1H), 1.41 (s, 9H), 1.22 (s, 9H). ^{13}C NMR (126 MHz, DMSO-*d*$_6$) δ 161.0, 144.9, 140.8, 137.1, 133.1, 129.1, 122.6, 120.2, 114.2, 94.5, 52.9, 32.5, 31.2, 30.7. ESI-MS (m/z): 369.2 (M+H$^+$).

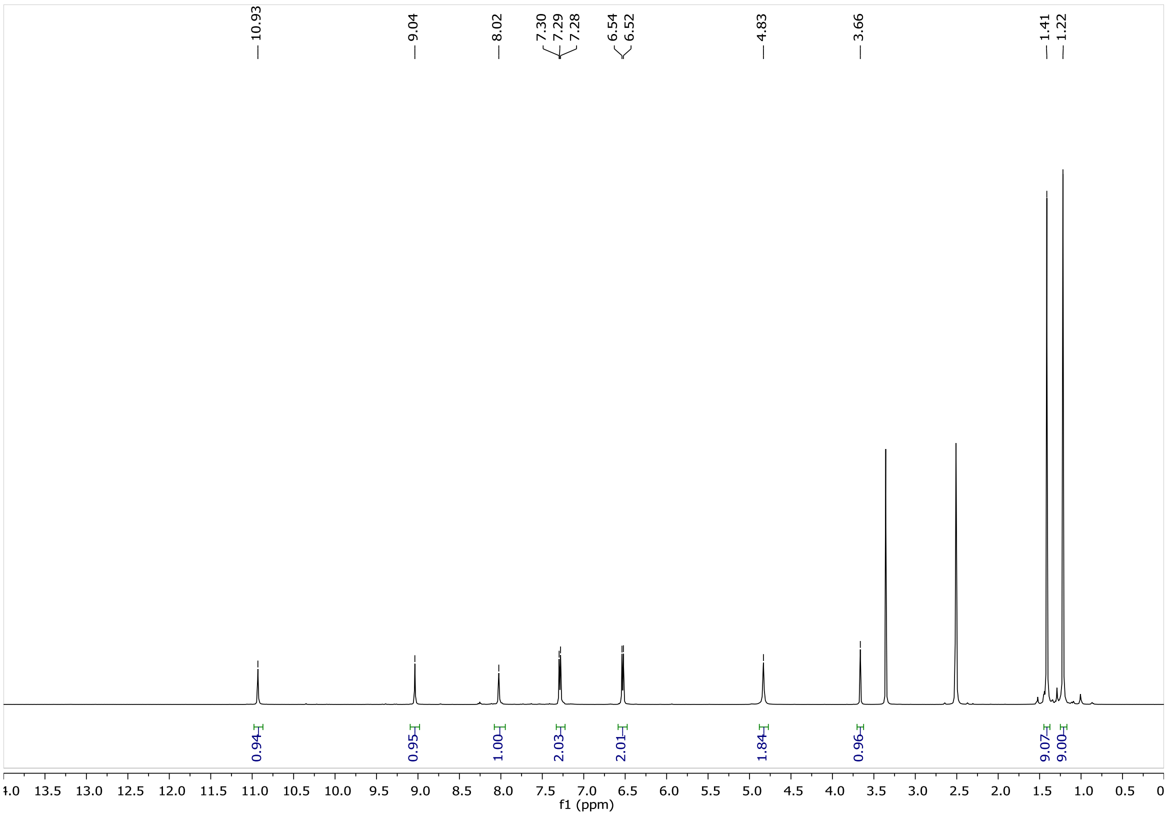

^{1}H NMR spectra of **DU325**

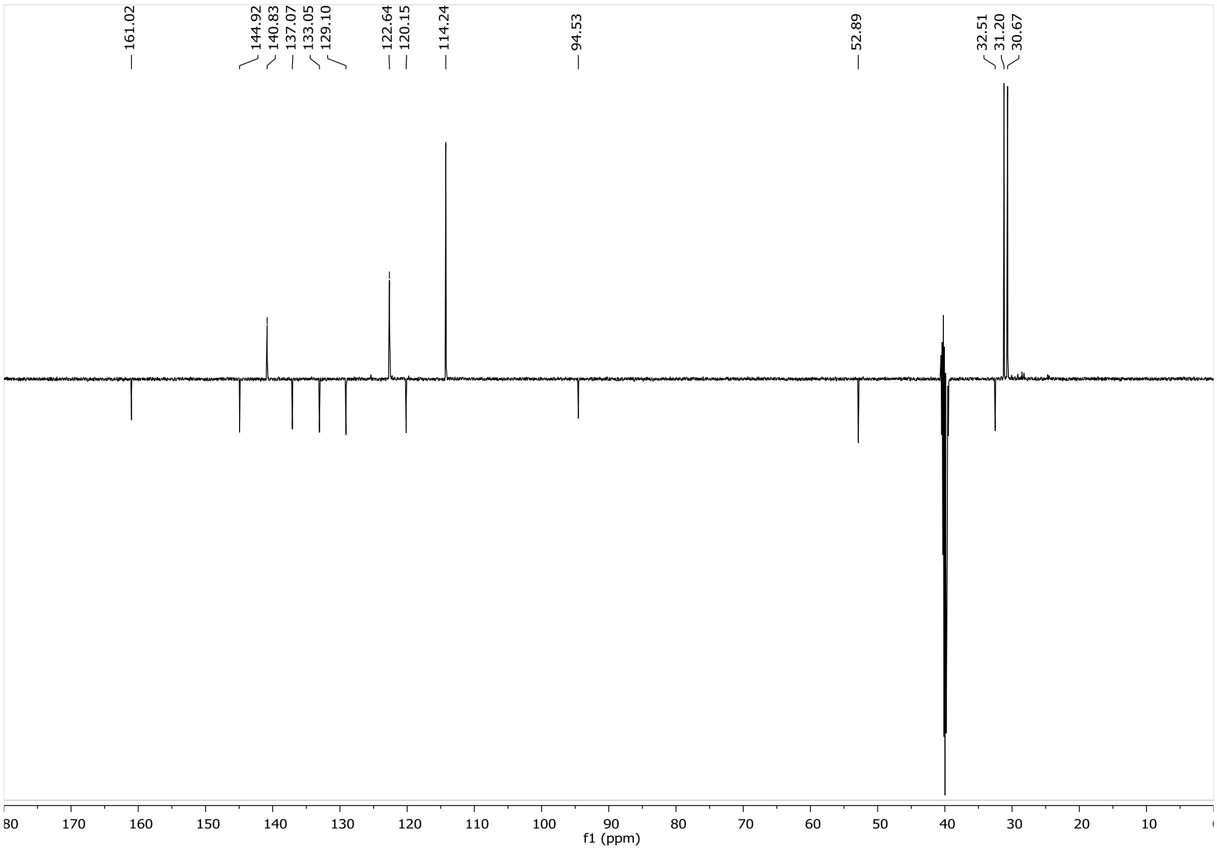

^{13}C NMR spectra of **DU325**

***2-(Tert-butyl)-3-(tert-butylamino)-N-(4-hydroxyphenyl)-1H-imidazo[1,2-b]pyrazole-7-carboxamide* (DU385)**

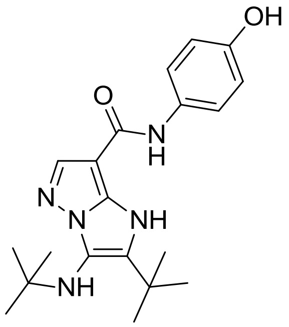

White solid; yield: 45%; m.p. 197 °C; ^{1}H NMR (500 MHz, DMSO-d_6) δ 10.98 (s, 1H), 9.19 (s, 1H), 9.13 (s, 1H), 8.06 (s, 1H), 7.45 (d, *J* = 8.3 Hz, 2H), 6.71 (d, *J* = 8.3 Hz, 2H), 3.67 (s, 1H), 1.42 (s, 9H), 1.22 (s, 9H). ^{13}C NMR (126 MHz, DMSO-d_6) δ 161.1, 153.4, 140.8, 137.2, 133.1, 131.7, 122.4, 120.2, 115.4, 94.4, 52.9, 32.5, 31.2, 30.8. ESI-MS (m/z): 370.2 (M+H$^+$).

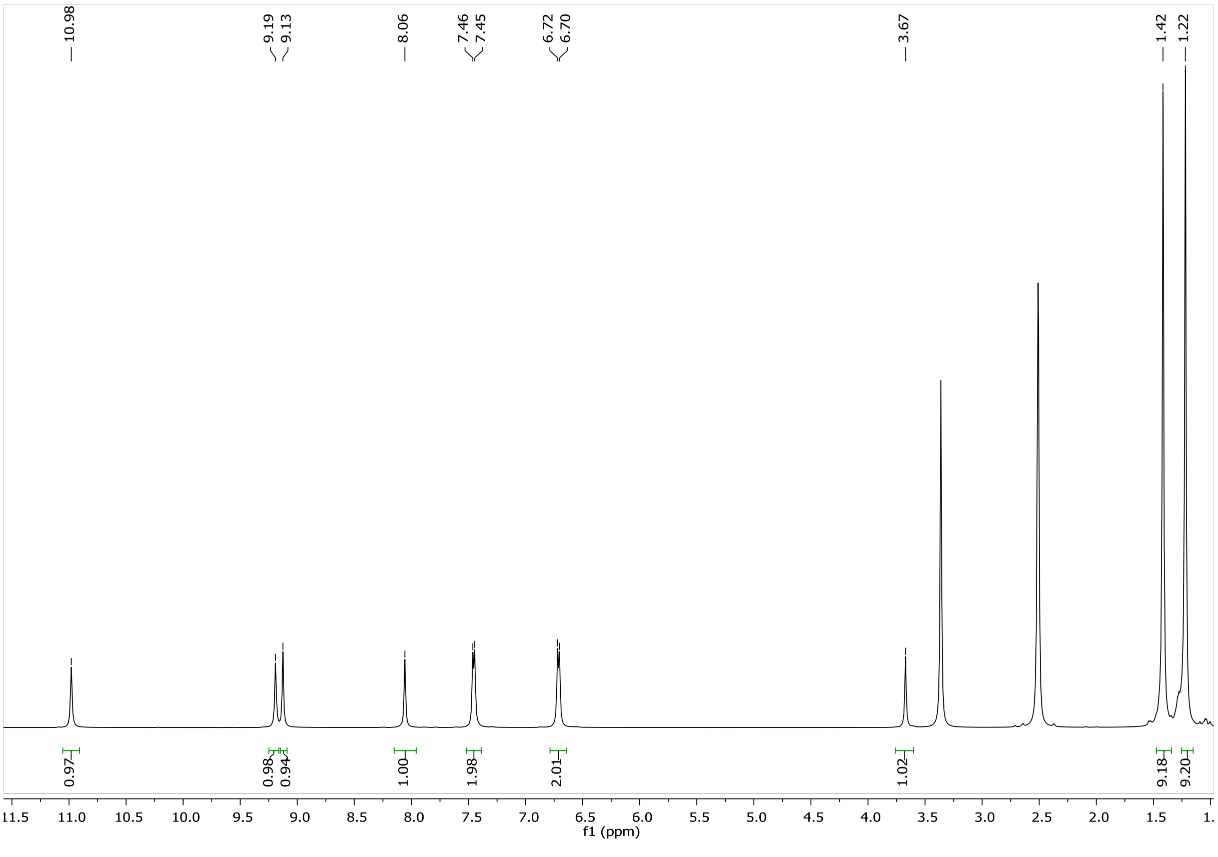

^{1}H NMR spectra of **DU385**

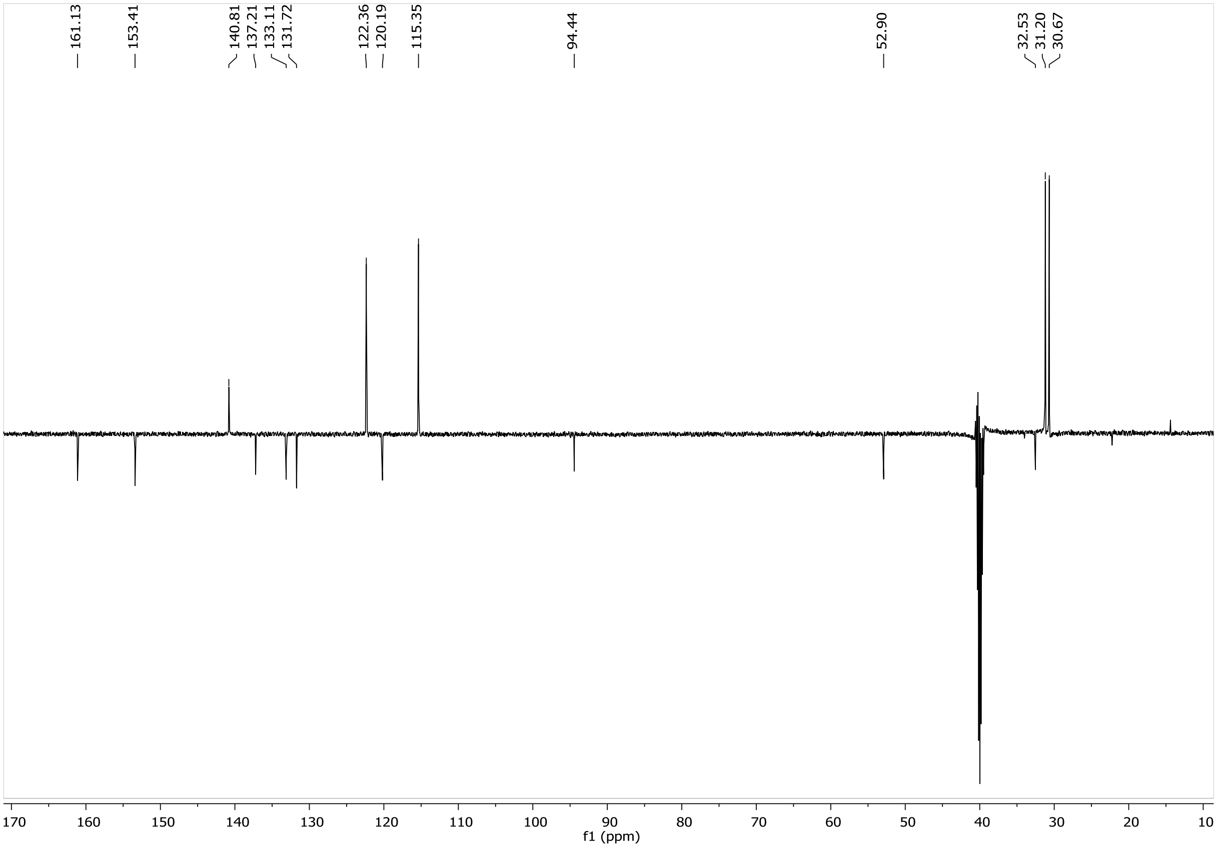

^{13}C NMR spectra of **DU385**

2-(Tert-butyl)-N-(4-hydroxyphenyl)-3-((2,4,4-trimethylpentan-2-yl)amino)-1H-imidazo[1,2-b]pyrazole-7-carboxamide **(DU441)**

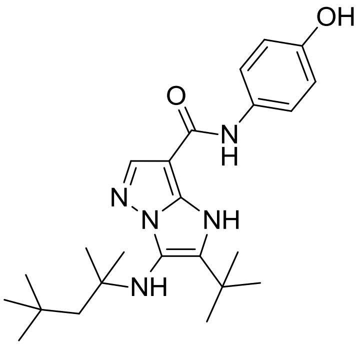

White solid; yield: 48%; m.p. 168 °C; ^{1}H NMR (500 MHz, DMSO-*d*$_6$) δ 10.98 (s, 1H), 9.18 (s, 1H), 9.13 (s, 1H), 8.05 (s, 1H), 7.45 (d, *J* = 8.4 Hz, 2H), 6.71 (d, *J* = 8.3 Hz, 2H), 3.51 (s, 1H), 1.69 (s, 2H), 1.42 (s, 9H), 1.25 (s, 6H), 1.04 (s, 9H). ^{13}C-NMR (126 MHz, DMSO-*d*$_6$) δ 161.1, 153.4, 140.8, 137.2, 133.1, 131.7, 122.4, 119.9, 115.4, 94.4, 56.9, 56.5, 32.5, 32.2, 31.9, 30.7, 30.1. ESI-MS (m/z): 426.2 (M+H$^+$).

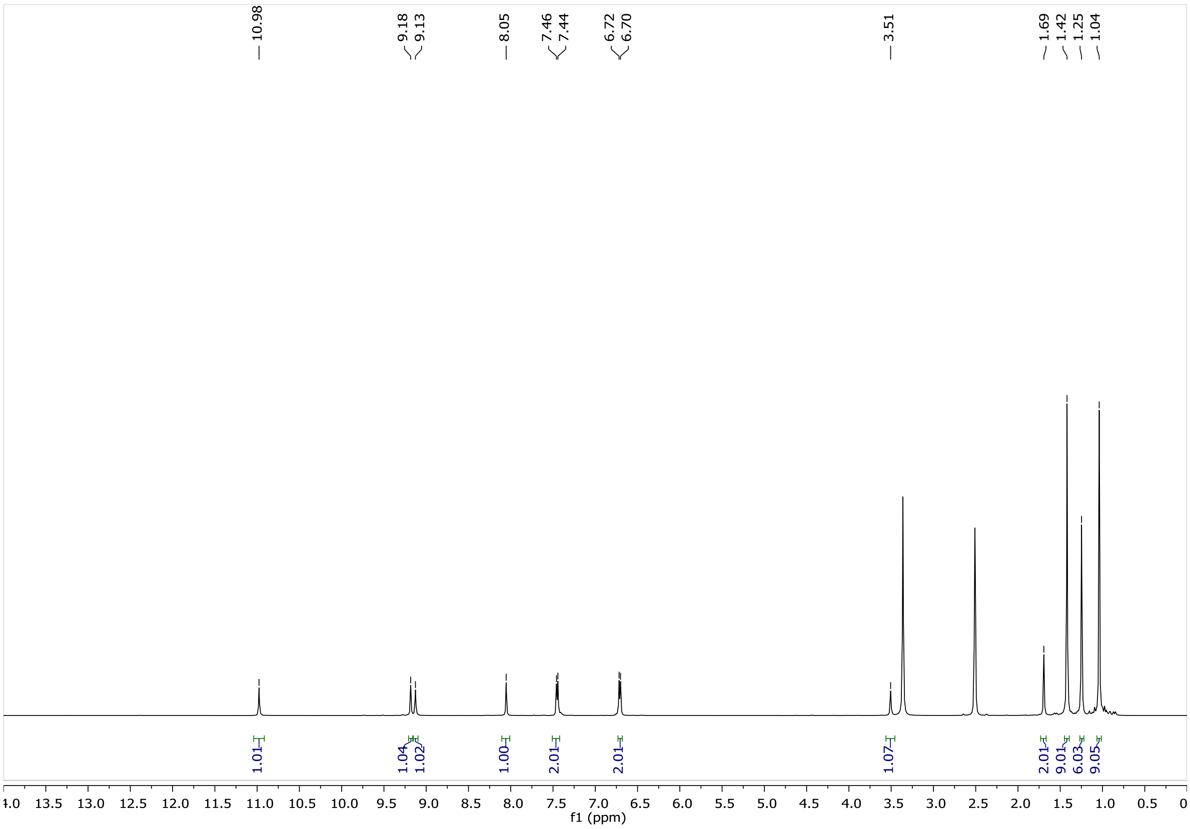

^{1}H NMR spectra of **DU441**

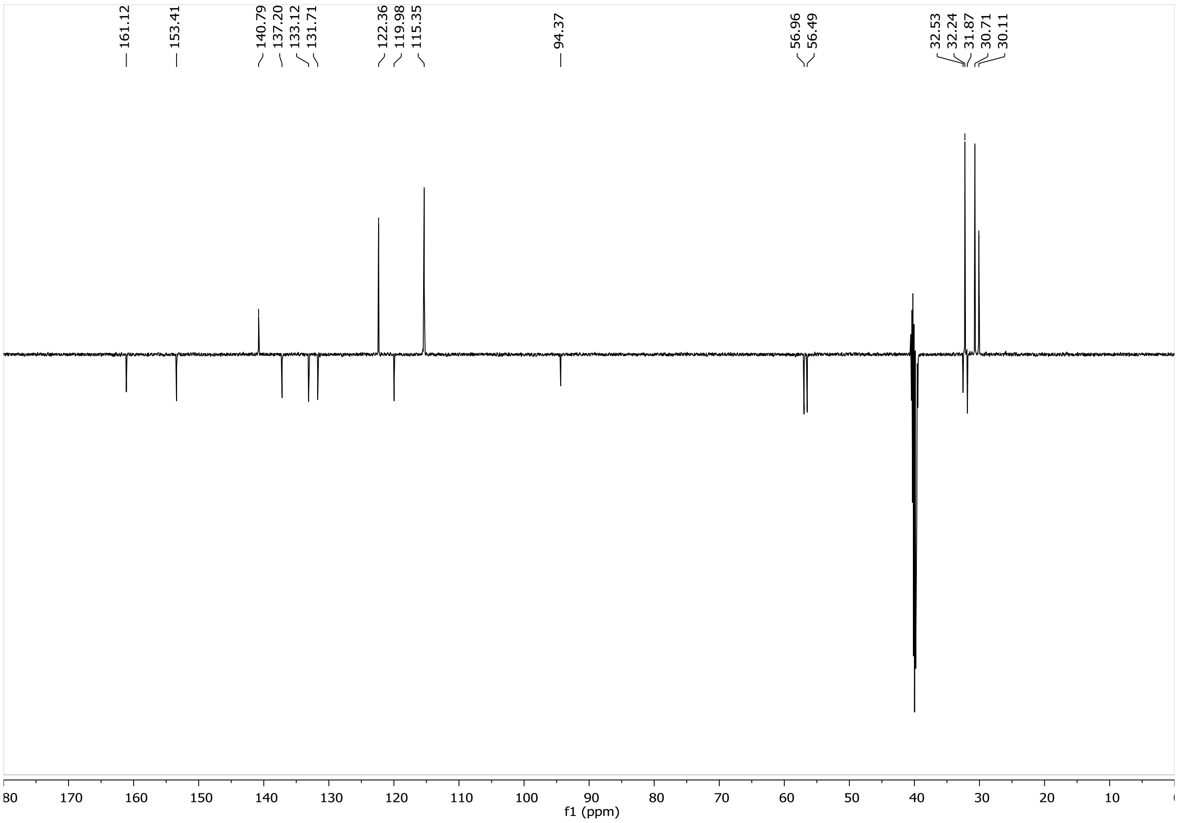

^{13}C NMR spectra of **DU441**

***2-(Tert-butyl)-3-(cyclohexylamino)-N-(4-hydroxyphenyl)-1H-imidazo[1,2-b]pyrazole-7-carbox-amide* (DU442)**

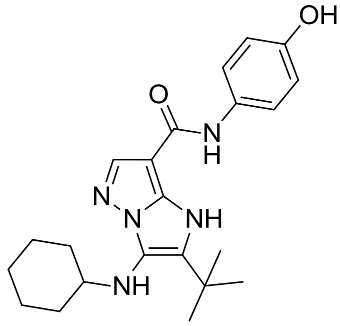

White solid; yield: 43%; m.p. 212 °C; ^{1}H NMR (500 MHz, DMSO-d_6) δ 10.96 (s, 1H), 9.20 (s, 1H), 9.13 (s, 1H), 8.08 (s, 1H), 7.46 (d, J = 8.4 Hz, 2H), 6.71 (d, J = 8.4 Hz, 2H), 3.91 (d, J = 4.7 Hz, 1H), 3.32 (s, 1H), 1.81–1.73 (m, 2H), 1.72–1.65 (m, 2H), 1.59–1.52 (m, 1H), 1.40 (s, 9H), 1.22–1.11 (m, 5H). ^{13}C-NMR (126 MHz, DMSO-d_6) δ 161.1, 153.4, 141.2, 137.1, 131.7, 130.4, 122.3, 121.1, 115.4, 94.6, 54.9, 33.7, 32.4, 30.5, 26.1, 25.0. ESI-MS (m/z): 396.2 (M+H^{+}).

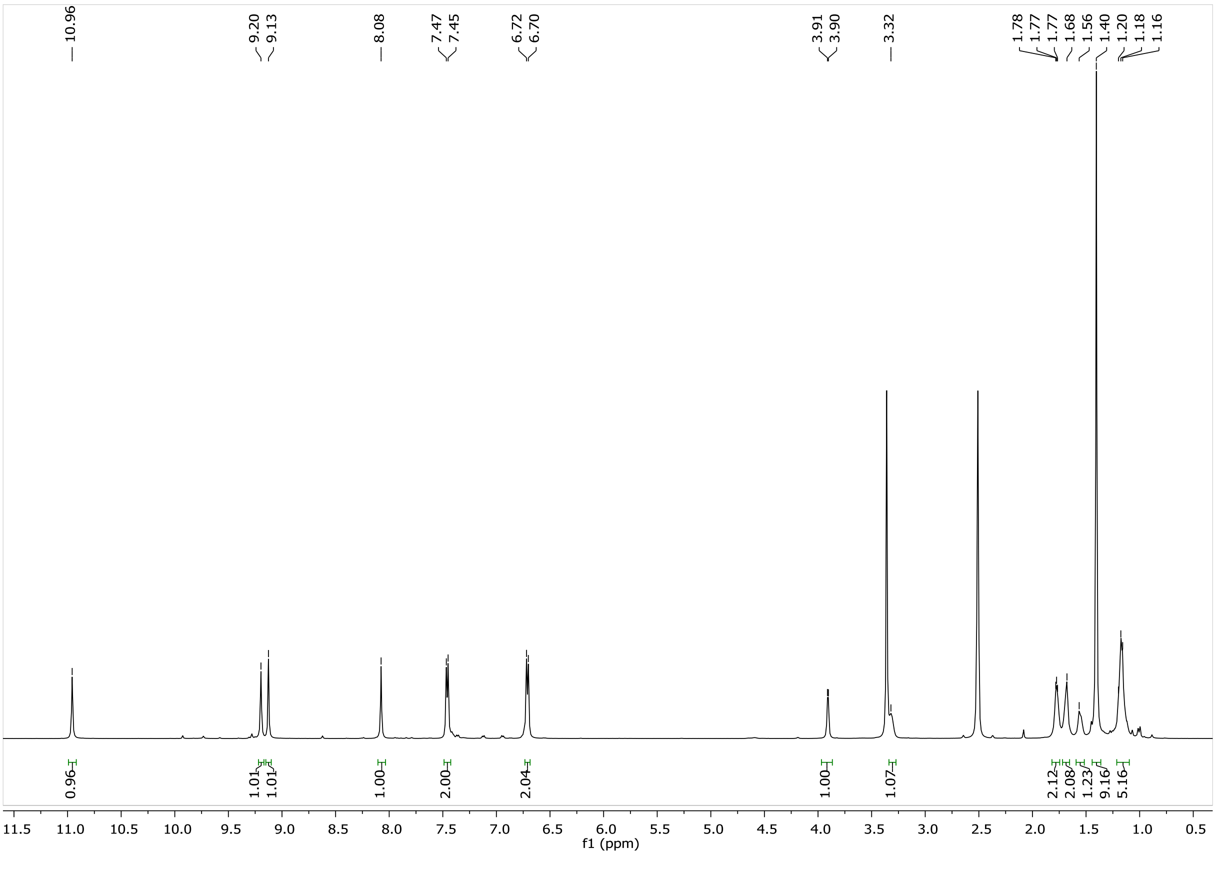

^{1}H NMR spectra of **DU442**

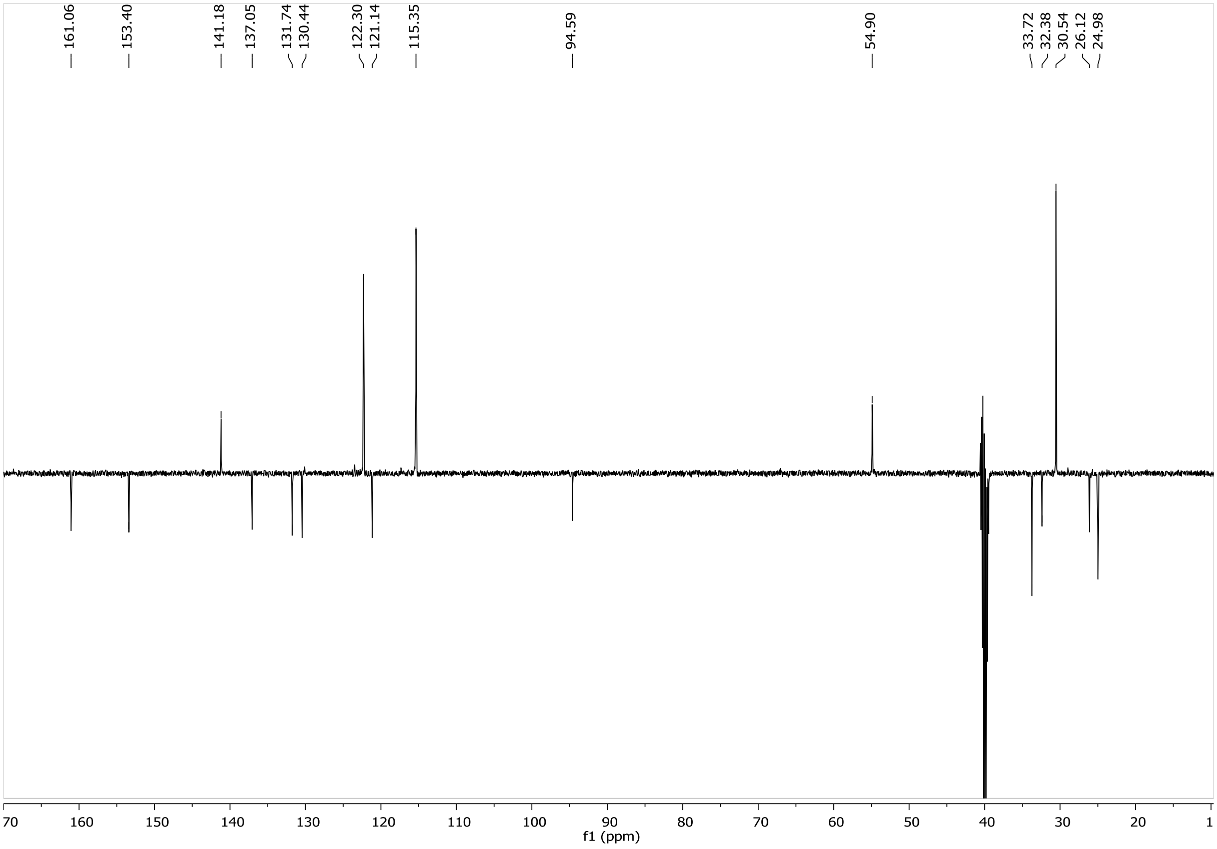

^{13}C NMR spectra of **DU442**

N-(4-aminophenyl)-2-(tert-butyl)-3-((2,4,4-trimethylpentan-2-yl)amino)-1H-imidazo[1,2-b]pyrazole-7-carboxamide **(DU443)**

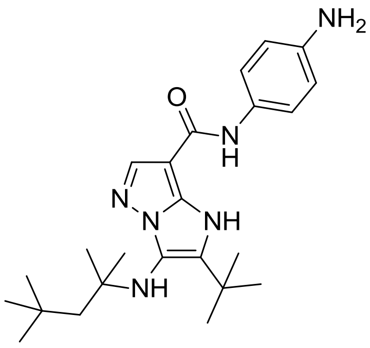

White solid; yield: 53%; m.p. 193 °C; ^{1}H NMR (500 MHz, DMSO-d_6) δ 10.93 (s, 1H), 9.03 (s, 1H), 8.02 (s, 1H), 7.29 (d, *J* = 8.7 Hz, 2H), 6.53 (d, *J* = 8.6 Hz, 2H), 4.83 (s, 2H), 3.50 (s, 1H), 1.69 (s, 2H), 1.42 (s, 9H), 1.25 (s, 6H), 1.04 (s, 9H). ^{13}C-NMR (126 MHz, DMSO-d_6) δ 161.0, 144.9, 140.8, 137.1, 133.1, 129.1, 122.6, 119.9, 114.2, 94.5, 56.9, 56.5, 32.5, 32.3, 31.9, 30.7, 30.1. ESI-MS (m/z): 425.2 (M+H^{+}).

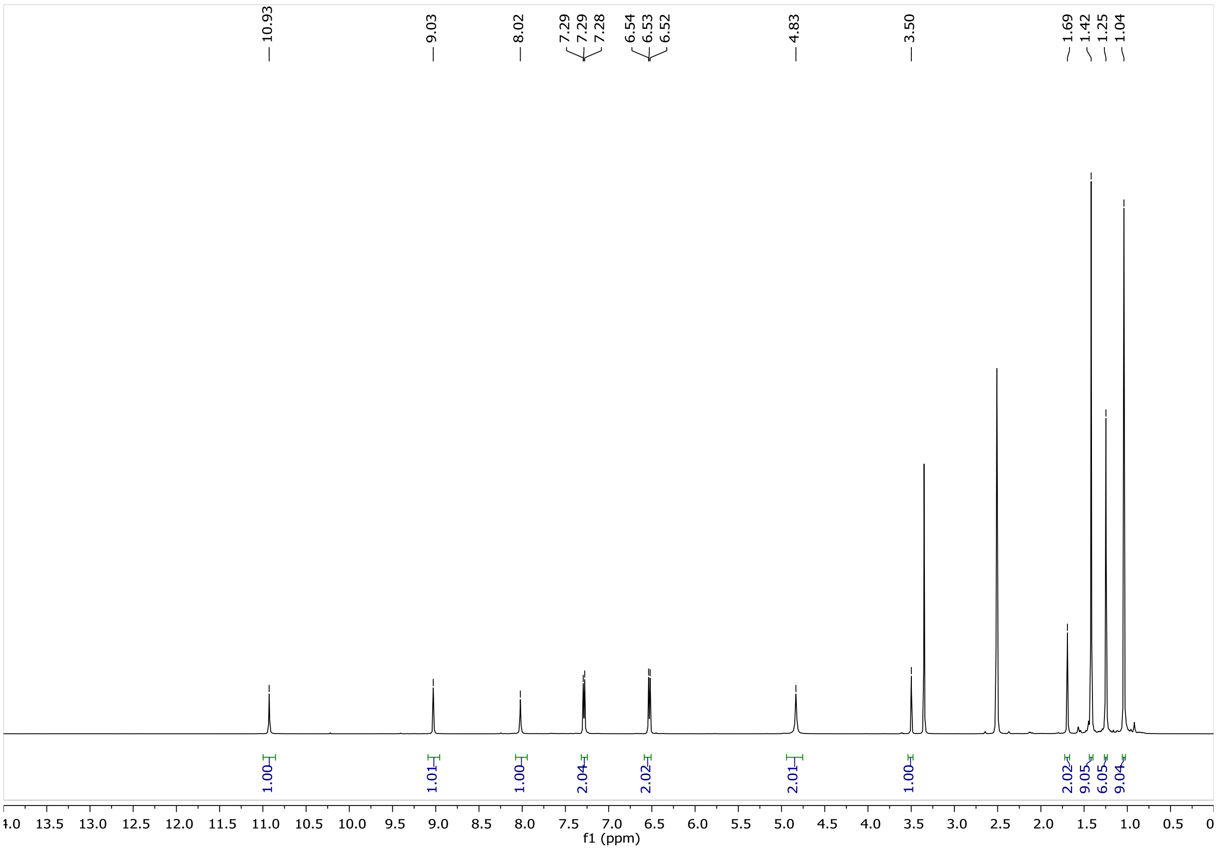

^{1}H NMR spectra of **DU443**

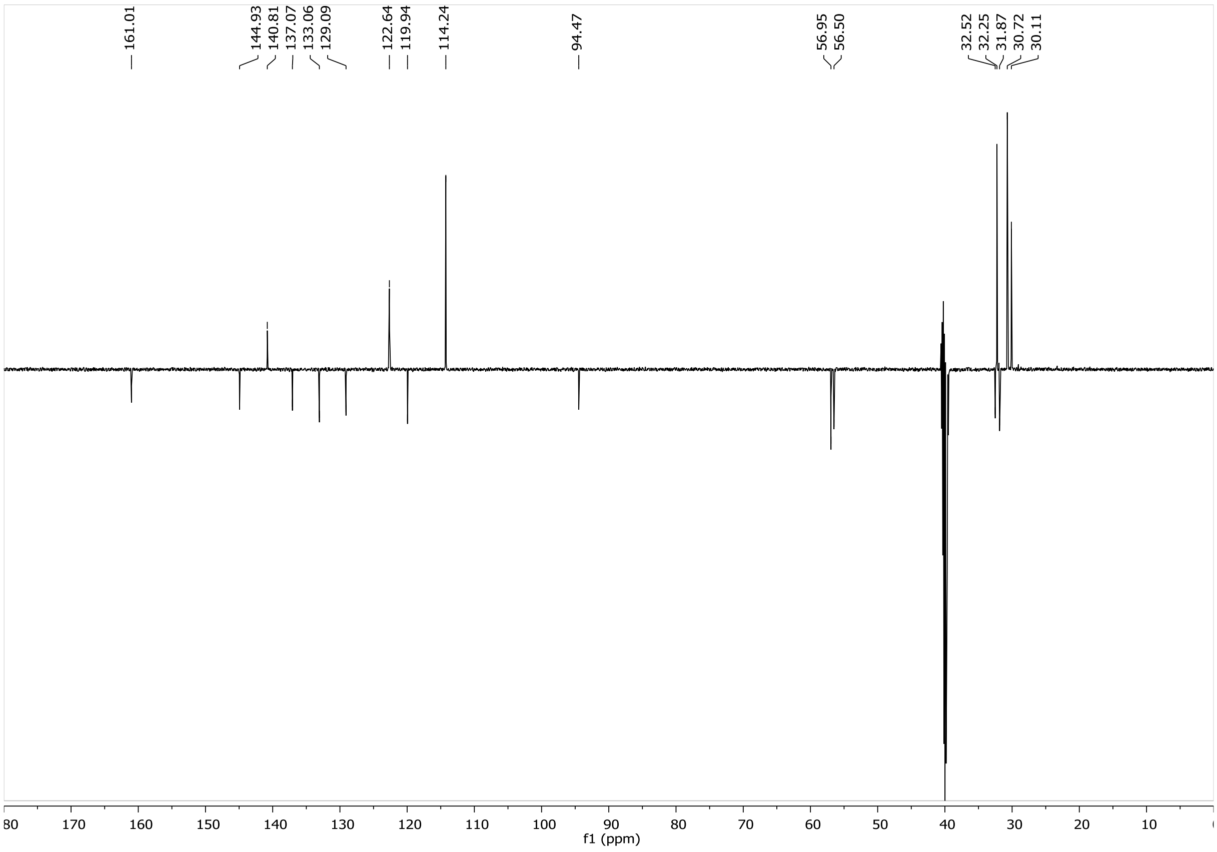

^{13}C NMR spectra of **DU443**

***2-(Tert-butyl)-N-(2-hydroxyphenyl)-3-((2,4,4-trimethylpentan-2-yl)amino)-1H-imidazo[1,2-b]pyrazole-7-carboxamide* (DU455)**

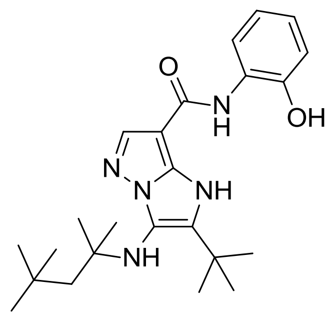

White solid; yield: 49%; m.p. 178 °C; ^{1}H NMR (500 MHz, DMSO-d_6) δ 11.14 (s, 1H), 10.05 (s, 1H), 9.19 (s, 1H), 8.13 (s, 1H), 7.49 (dd, *J* = 8.0, 1.6 Hz, 1H), 7.01 (td, *J* = 7.7, 1.7 Hz, 1H), 6.91 (dd, *J* = 8.1, 1.5 Hz, 1H), 6.83 (td, *J* = 7.6, 1.5 Hz, 1H), 3.57 (s, 1H), 1.70 (s, 2H), 1.43 (s, 9H), 1.25 (s, 6H), 1.04 (s, 9H). ^{13}C-NMR (126 MHz, DMSO-d_6) δ 162.3, 149.5, 141.4, 137.1, 133.3, 127.2, 125.6, 123.9, 120.3, 119.7, 117.7, 93.4, 56.9, 56.5, 32.6, 32.2, 31.9, 30.7, 30.1. ESI-MS (m/z): 426.2 (M+H+).

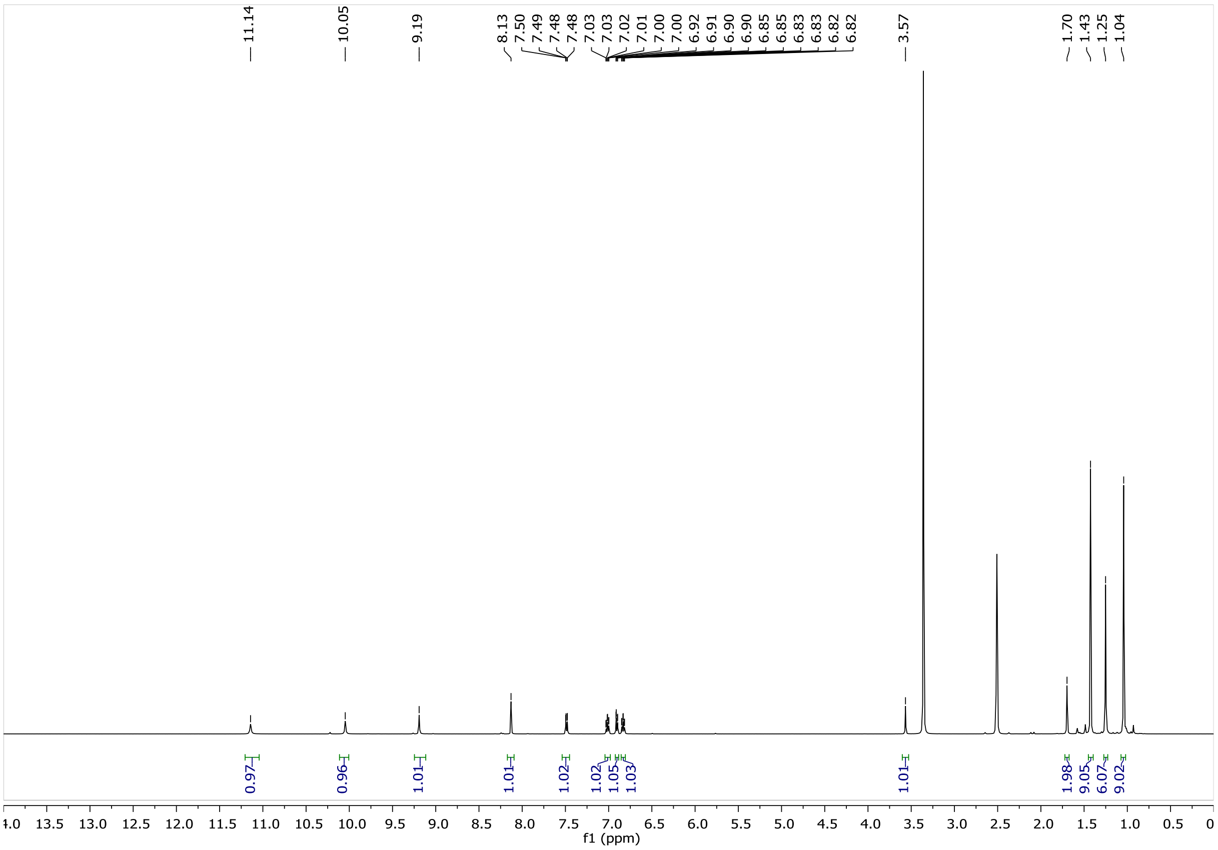

^{1}H NMR spectra of **DU455**

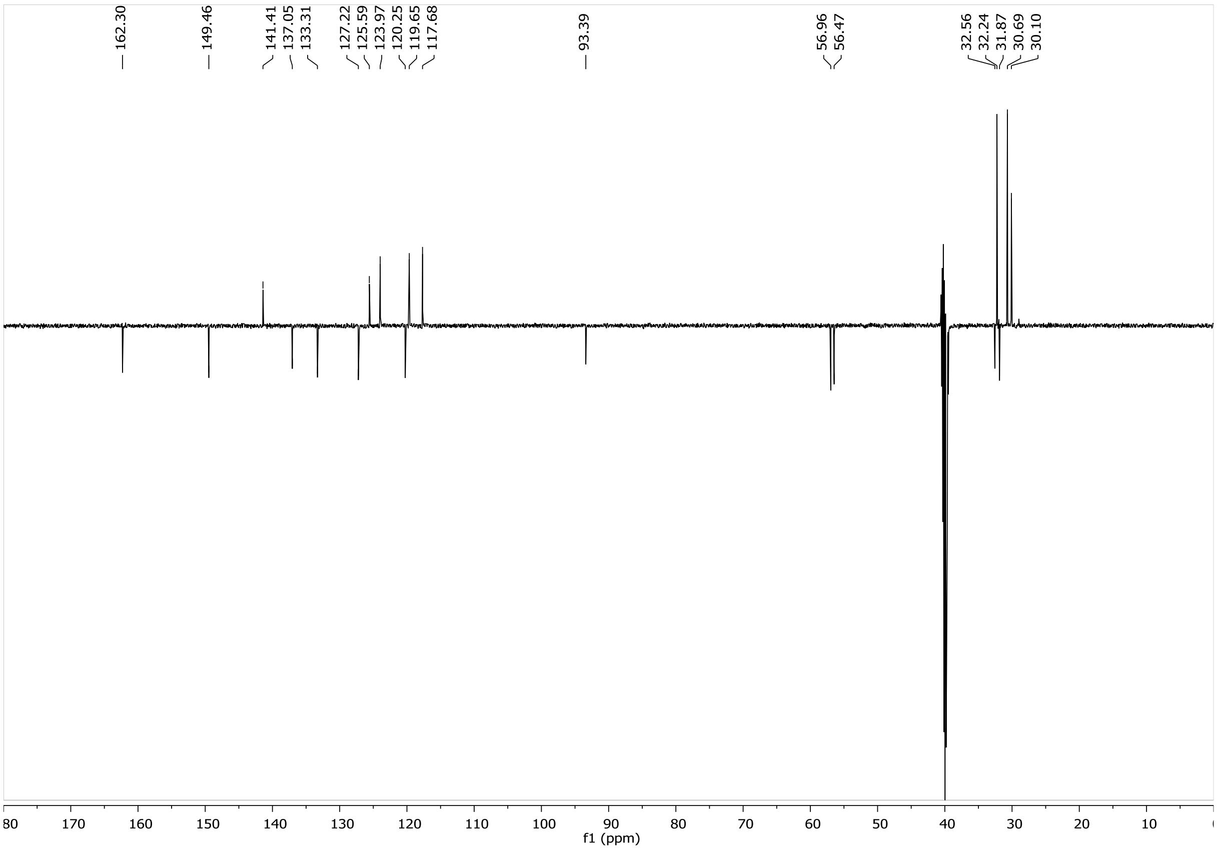

^{13}C NMR spectra of **DU455**

***2-(tert-butyl)-N-(3-hydroxyphenyl)-3-((2,4,4-trimethylpentan-2-yl)amino)-1H-imidazo[1,2-b]pyrazole-7-carboxamide* (DU456)**

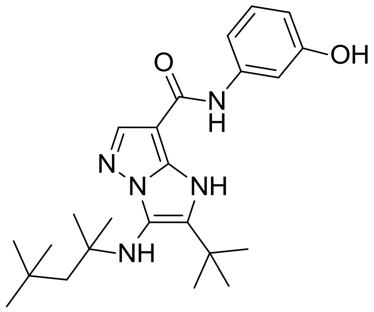

White solid; yield: 51%; m.p. 200 °C; ^{1}H NMR (500 MHz, DMSO-d_6) δ 11.05 (s, 1H), 9.30 (s, 1H), 9.28 (s, 1H), 8.12 (s, 1H), 7.25 (t, J = 2.2 Hz, 1H), 7.14 (d, J = 8.2 Hz, 1H), 7.07 (d, J = 8.1 Hz, 1H), 6.43 (d, J = 9.1 Hz, 1H), 3.52 (s, 1H), 1.70 (s, 2H), 1.42 (s, 9H), 1.25 (s, 6H), 1.04 (s, 9H). ^{13}C-NMR (126 MHz, DMSO-d_6) δ 161.4, 157.9, 141.4, 140.8, 137.4, 133.2, 129.5, 120.0, 111.0, 110.1, 107.4, 94.5, 56.9, 56.5, 32.5, 32.2, 31.9, 30.7, 30.1. ESI-MS (m/z): 426.2 (M+H^{+}).

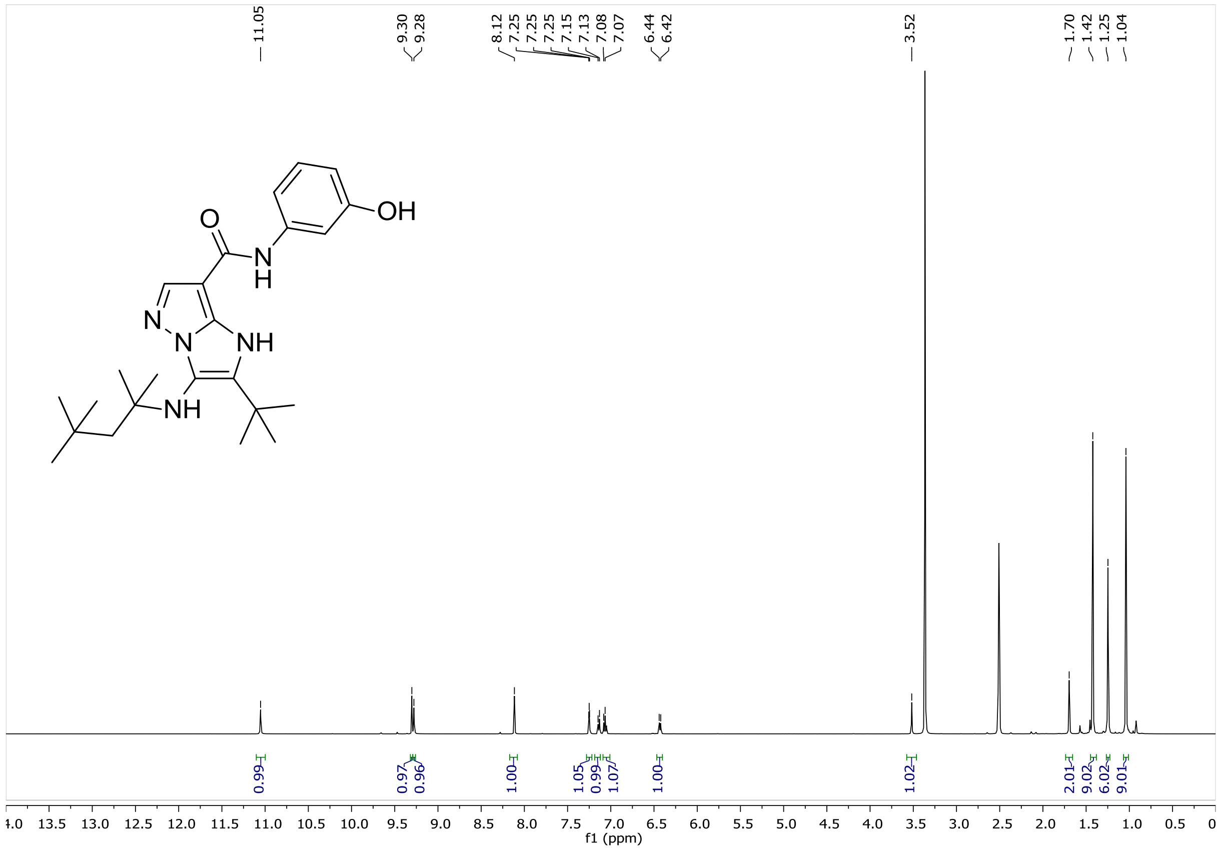

^{1}H NMR spectra of **DU456**

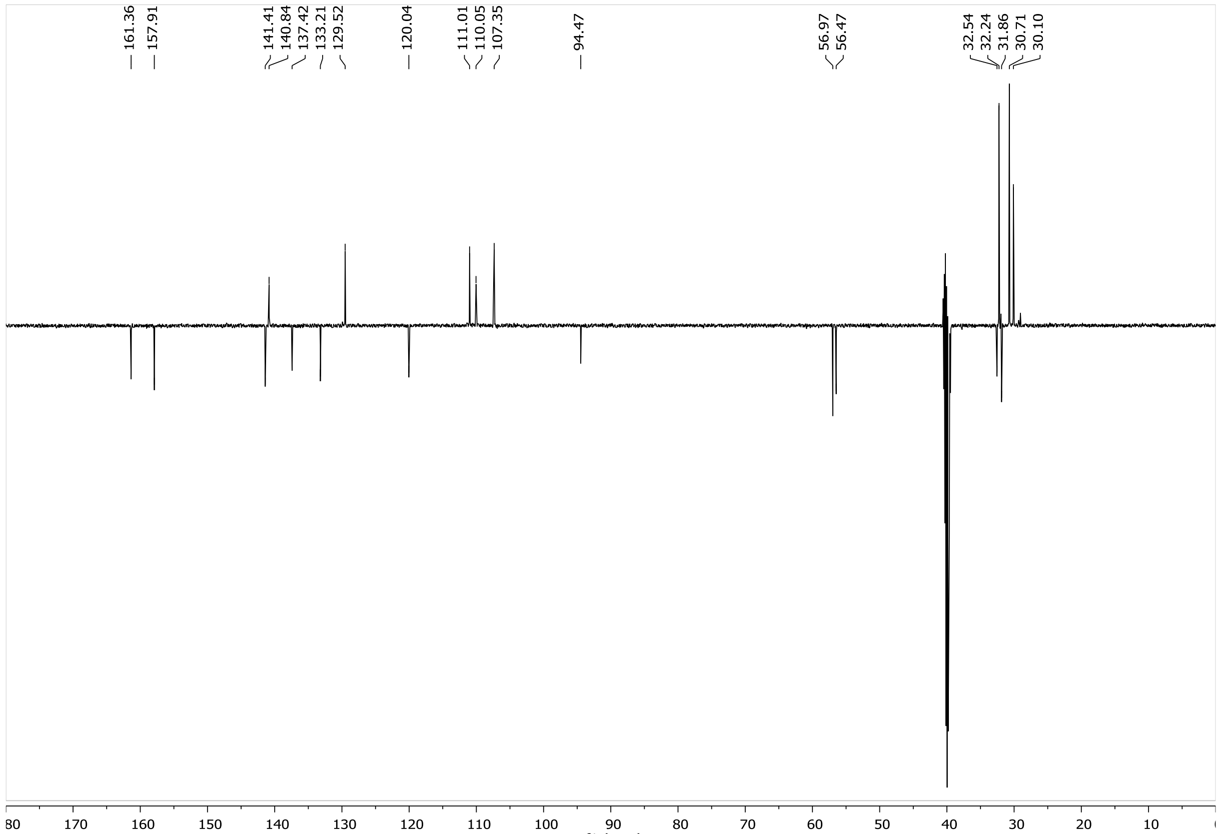

^{13}C NMR spectra of **DU456**
