# Supplementary material for: Imidazo[1,2-b]pyrazole-7-carboxamides Induce Apoptosis in Human Leukemia Cells at Nanomolar Concentrations
Source: Molecules. 2018 Nov 1;23(11):2845. doi: 10.3390/molecules23112845 (PMC6278434; doi:10.3390/molecules23112845)

Figure S8. Dose-response curves of imidazo[1,2-*b*]pyrazole-7-carboxamides on murine (4T1) and human (MCF7) breast cancer cells grown as a monolayer (2D) or spheroid (3D)

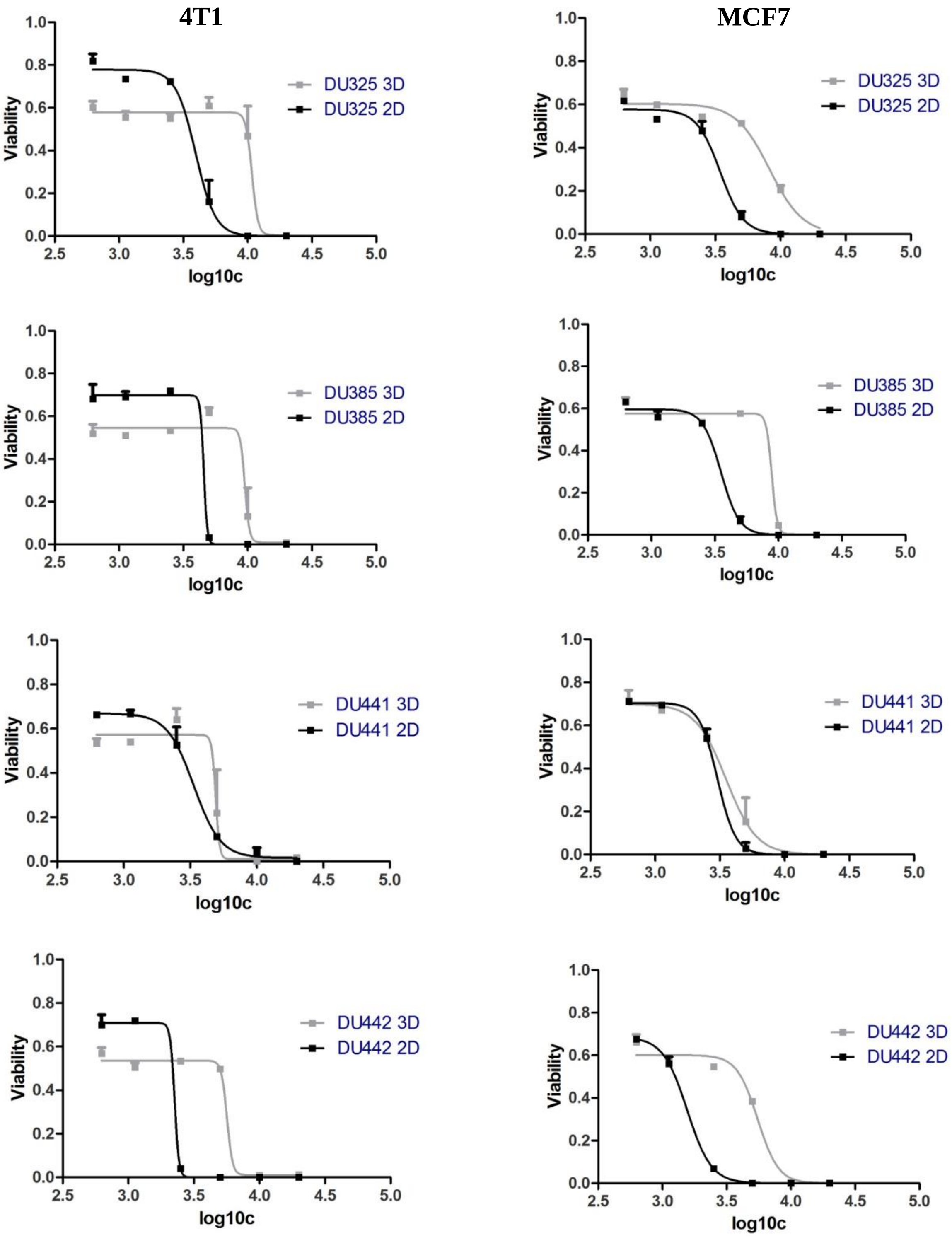

Supplement: Supplementary file 1 [file molecules-23-02845-s001.zip › molecules-372705-SI/Supplement Revised/FigureS8.pdf]
