# Supplementary figures and images for: Imidazo[1,2-b]pyrazole-7-carboxamides Induce Apoptosis in Human Leukemia Cells at Nanomolar Concentrations
Source: Molecules. 2018 Nov 1;23(11):2845. doi: 10.3390/molecules23112845 (PMC6278434; doi:10.3390/molecules23112845)

Figure S10. Detection of phosphatidylserine exposure on HL-60 cells

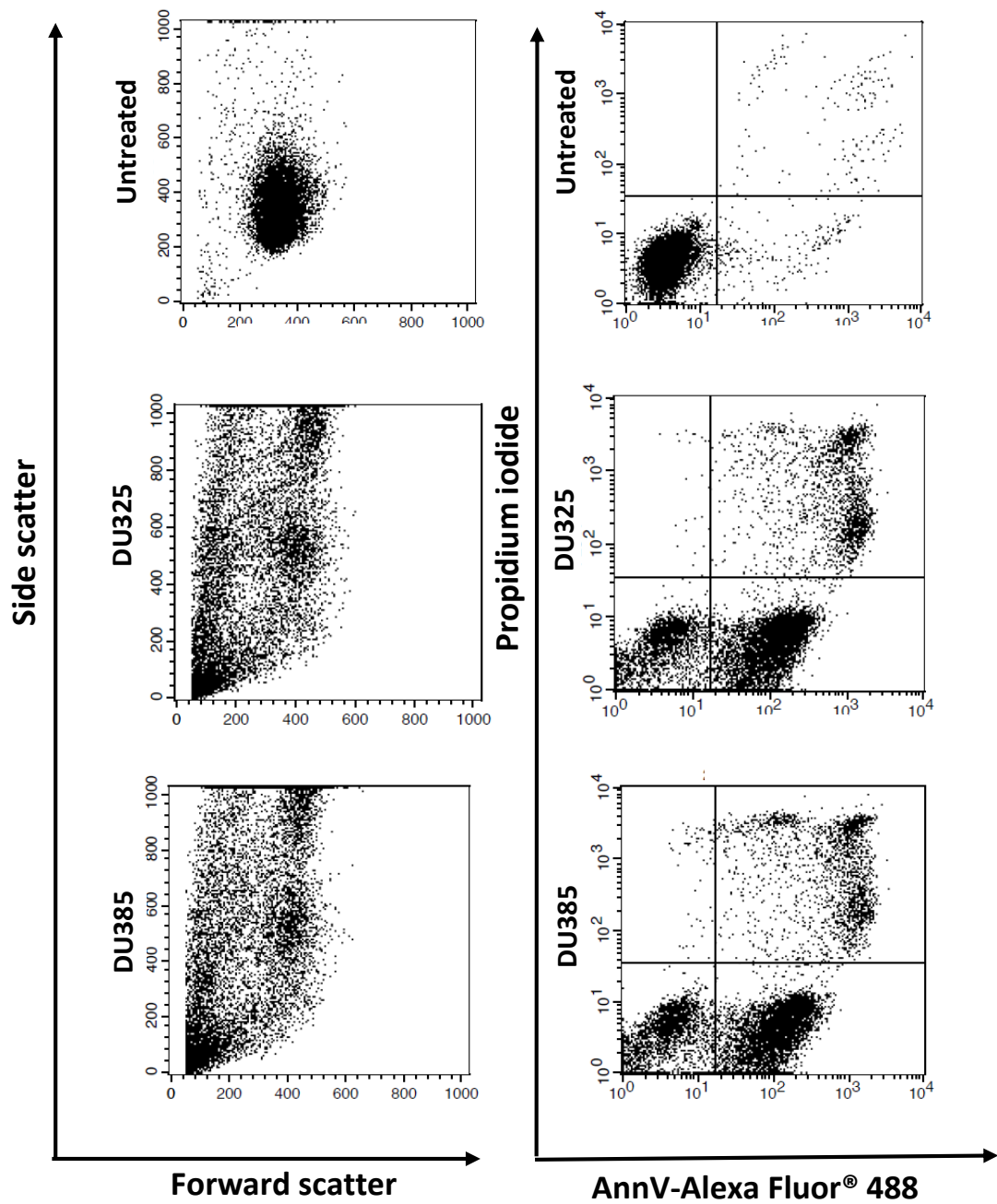

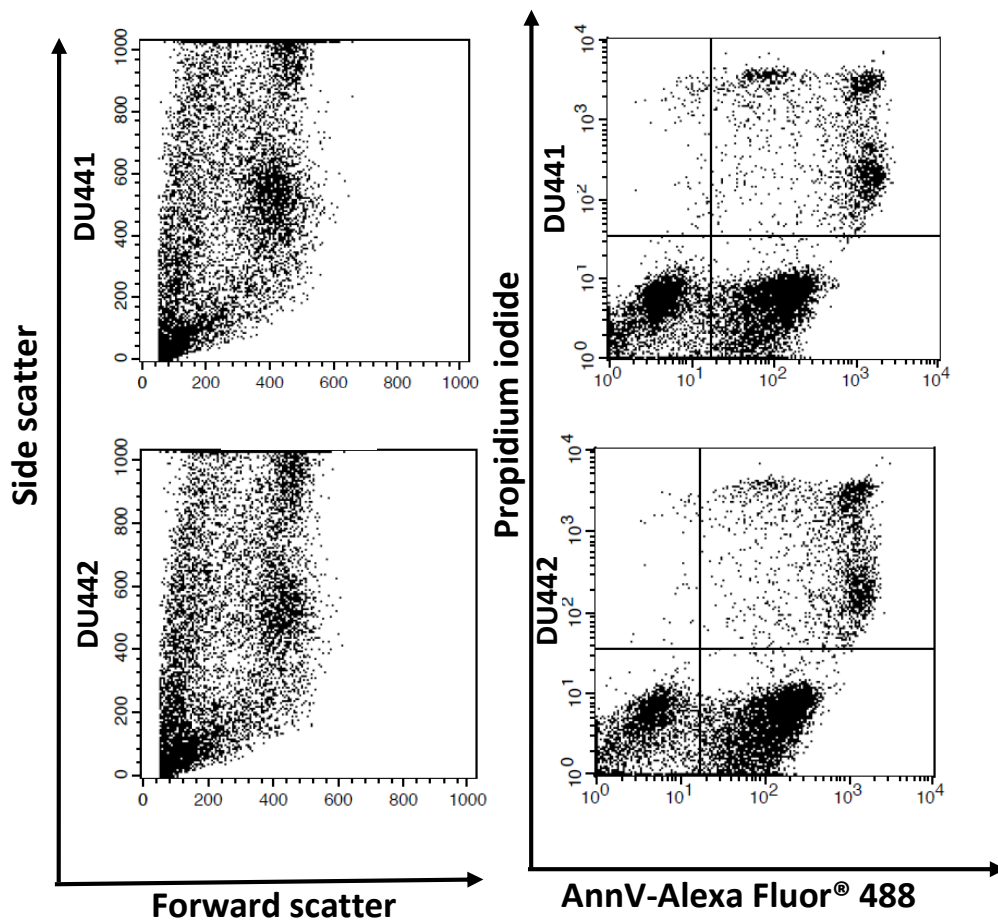

Supplement: Supplementary file 1 [file molecules-23-02845-s001.zip › molecules-372705-SI/Supplement Revised/FigureS10.pdf]

Figure S11. Detection of phosphatidylserine exposure on MOLT-4 cells

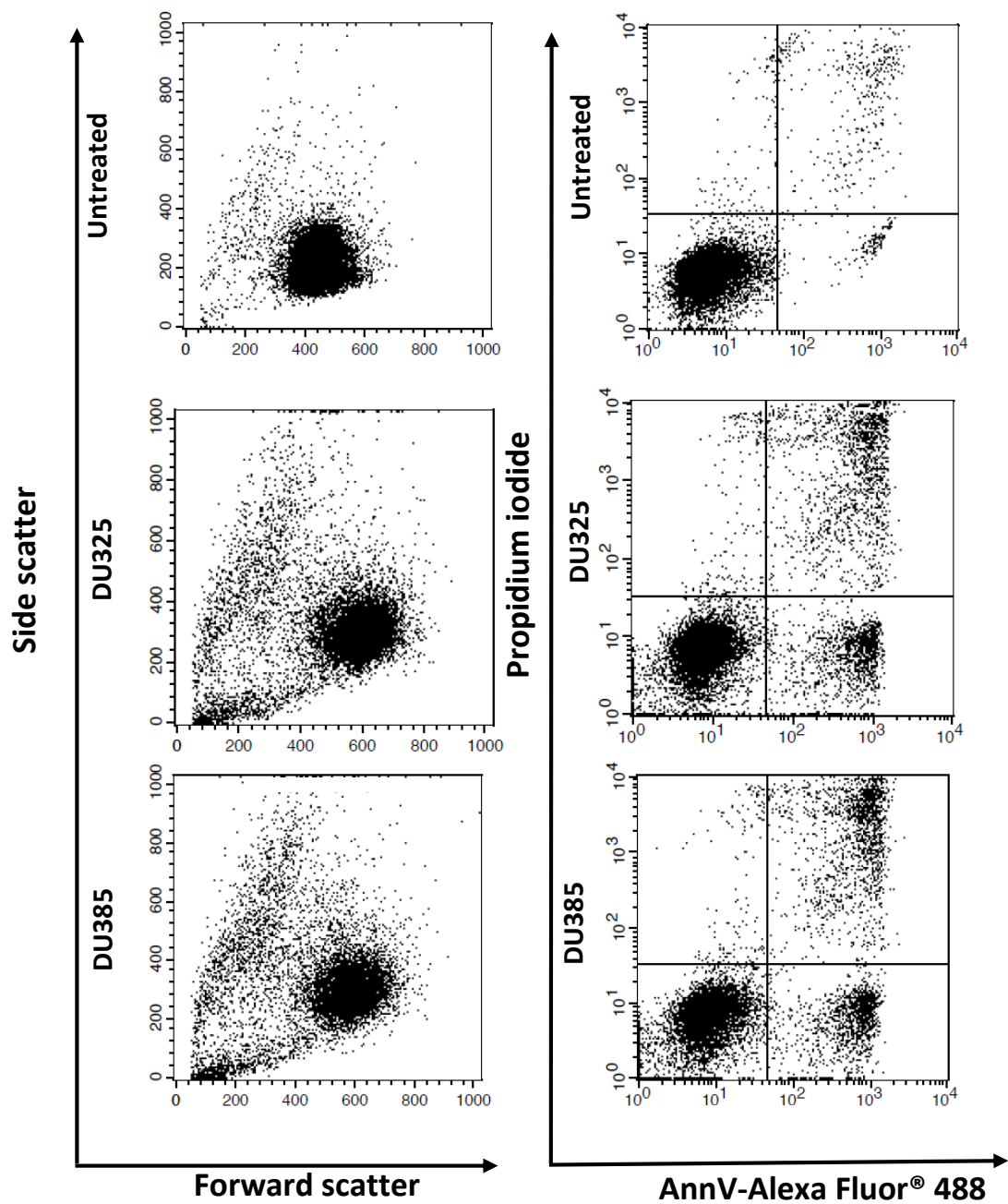

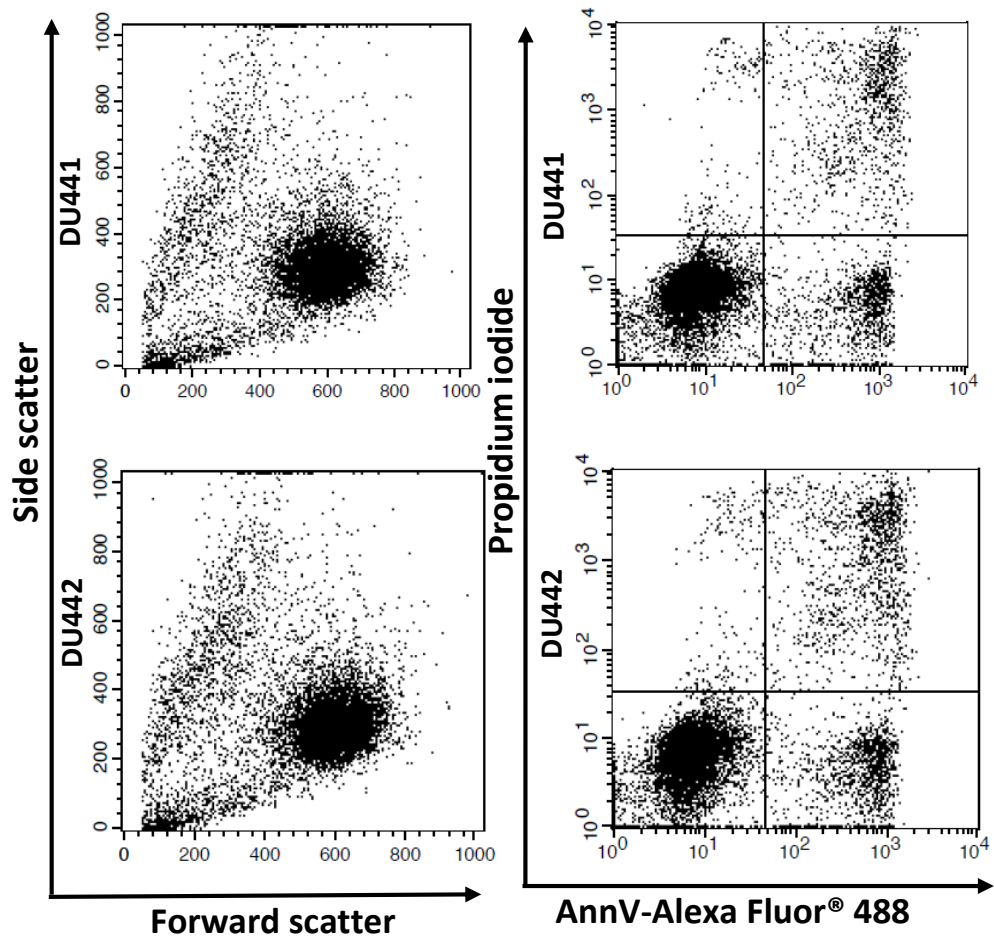

Supplement: Supplementary file 1 [file molecules-23-02845-s001.zip › molecules-372705-SI/Supplement Revised/FigureS11.pdf]

**Figure S12. Detection of the mitochondrial membrane potential**

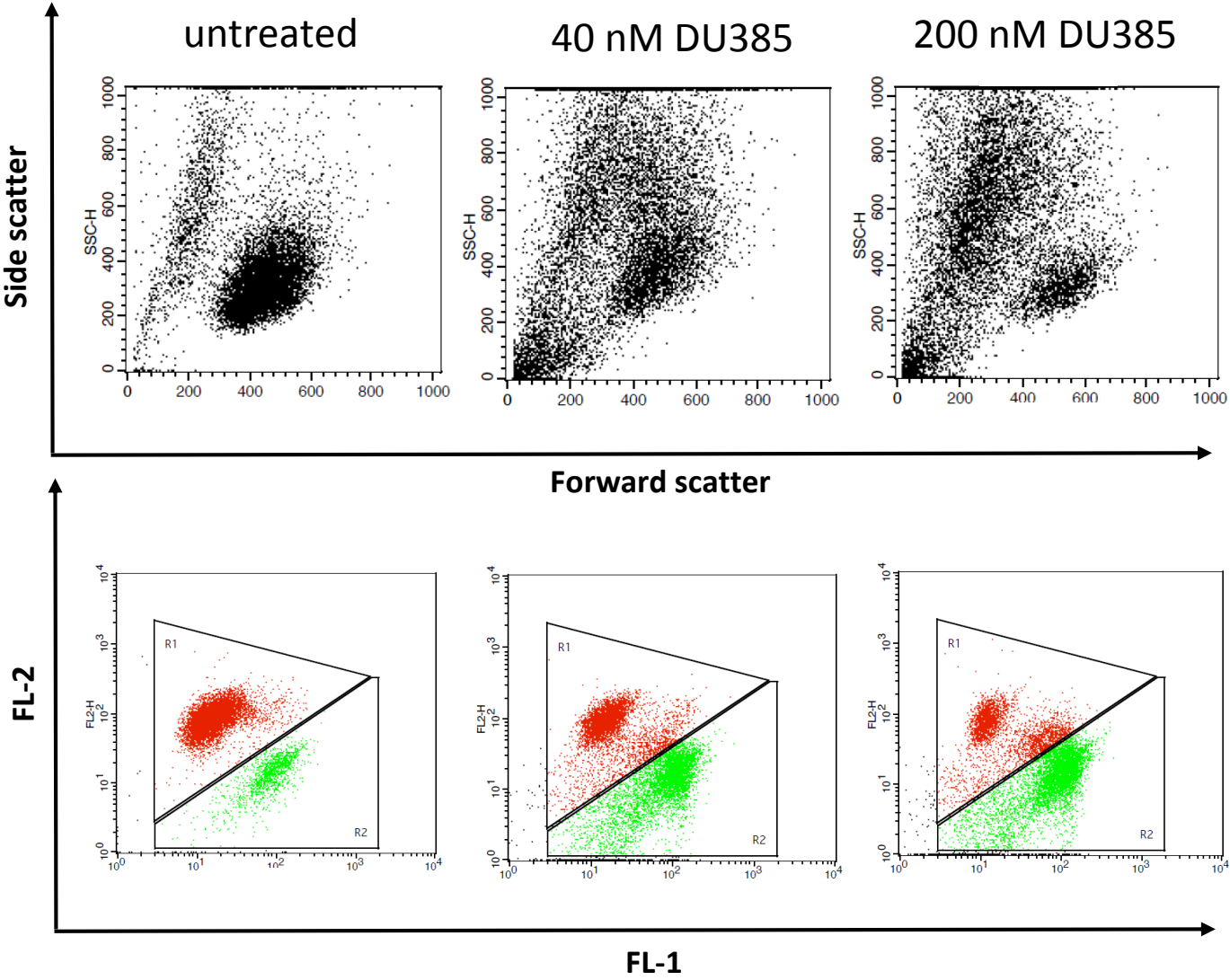

Supplement: Supplementary file 1 [file molecules-23-02845-s001.zip › molecules-372705-SI/Supplement Revised/FigureS12.pdf]

**Figure S2. Dose-response curves of imidazo[1,2-*b*]pyrazole-7-carboxamides on HL-60 cells**

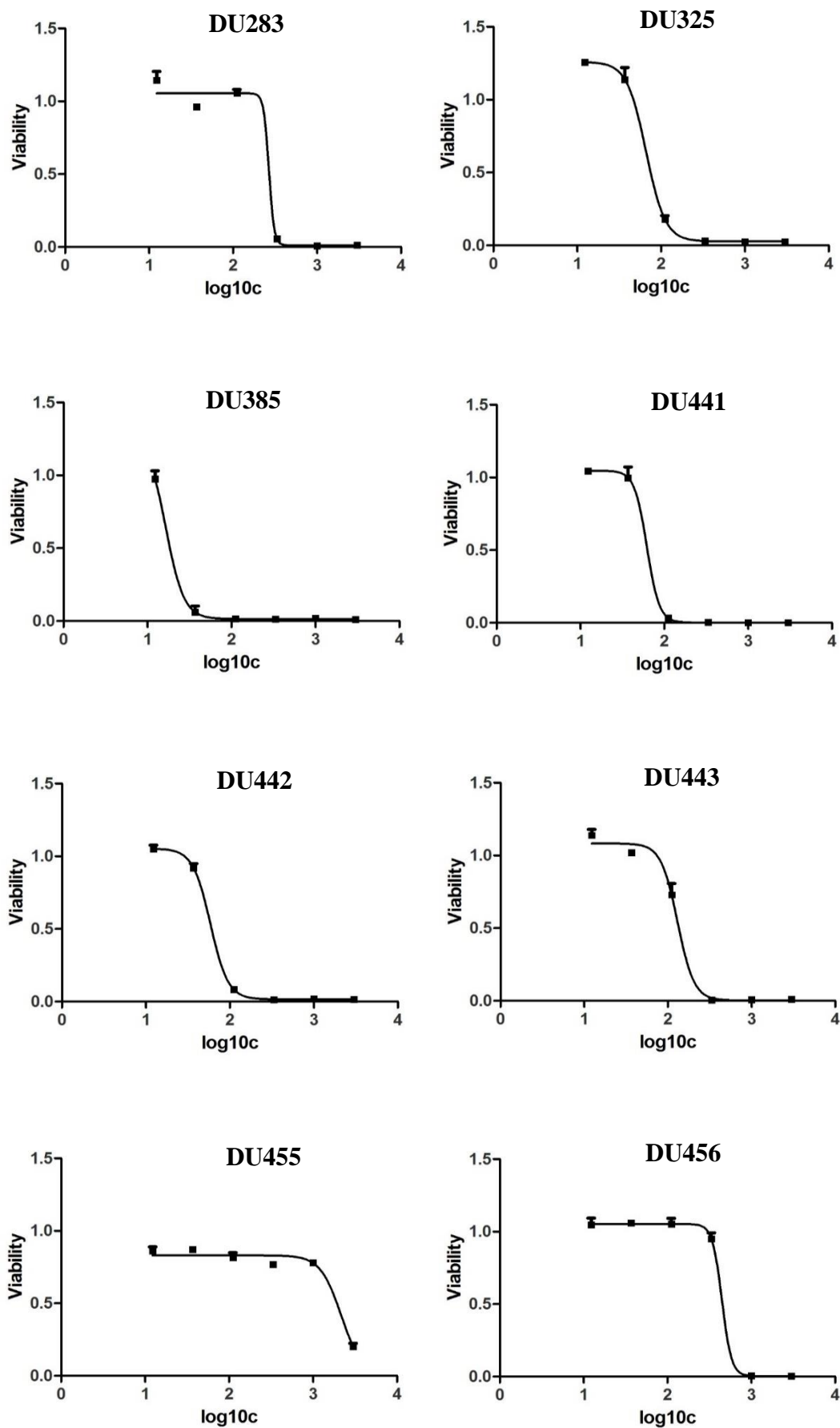

Supplement: Supplementary file 1 [file molecules-23-02845-s001.zip › molecules-372705-SI/Supplement Revised/FigureS2.pdf]

**Figure S3. Dose-response curves of imidazo[1,2-*b*]pyrazole-7-carboxamides on MOLT-4 cells**

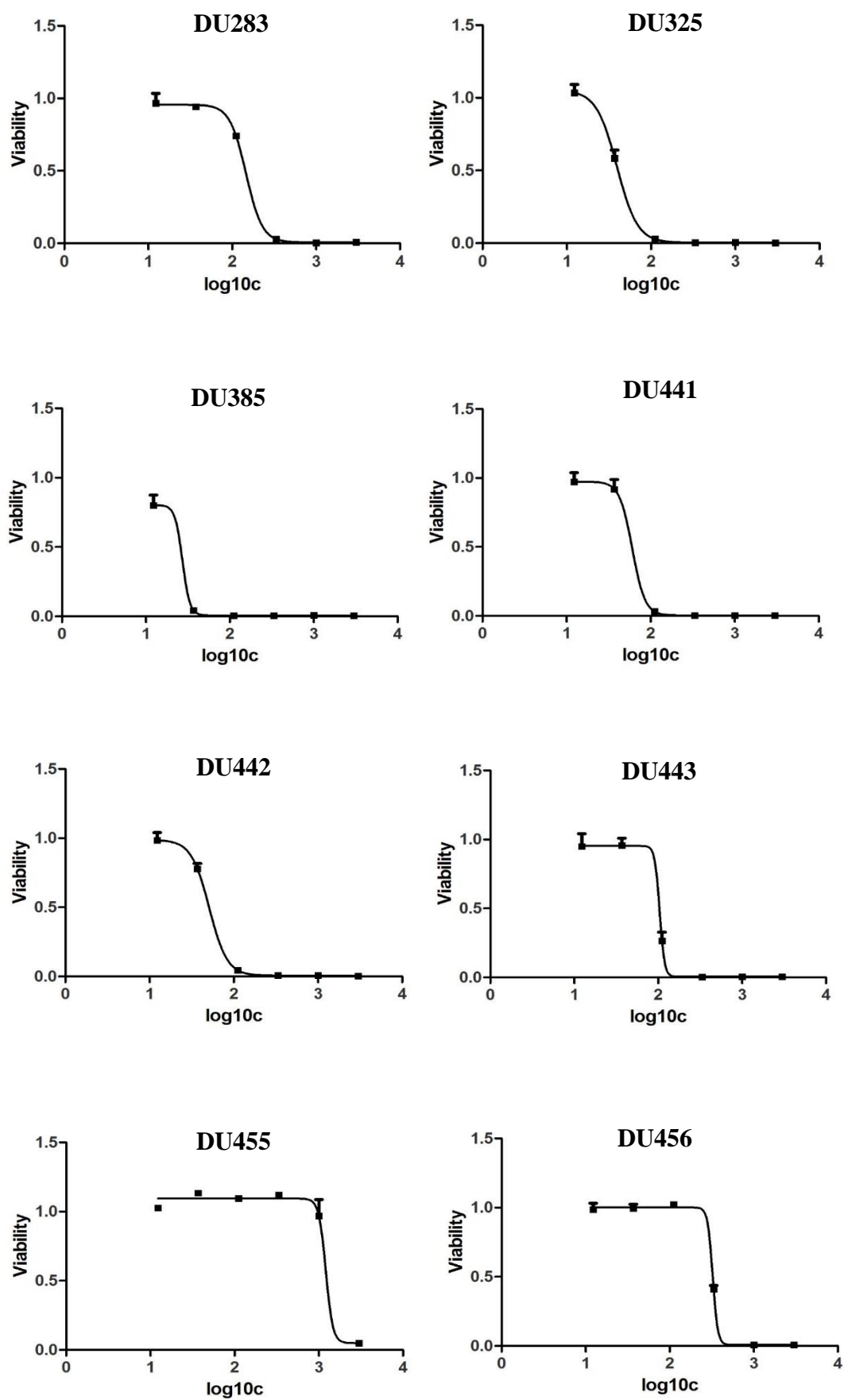

Supplement: Supplementary file 1 [file molecules-23-02845-s001.zip › molecules-372705-SI/Supplement Revised/FigureS3.pdf]

**Figure S4. Dose-response curves of imidazo[1,2-*b*]pyrazole-7-carboxamides on MV-4-11-cells**

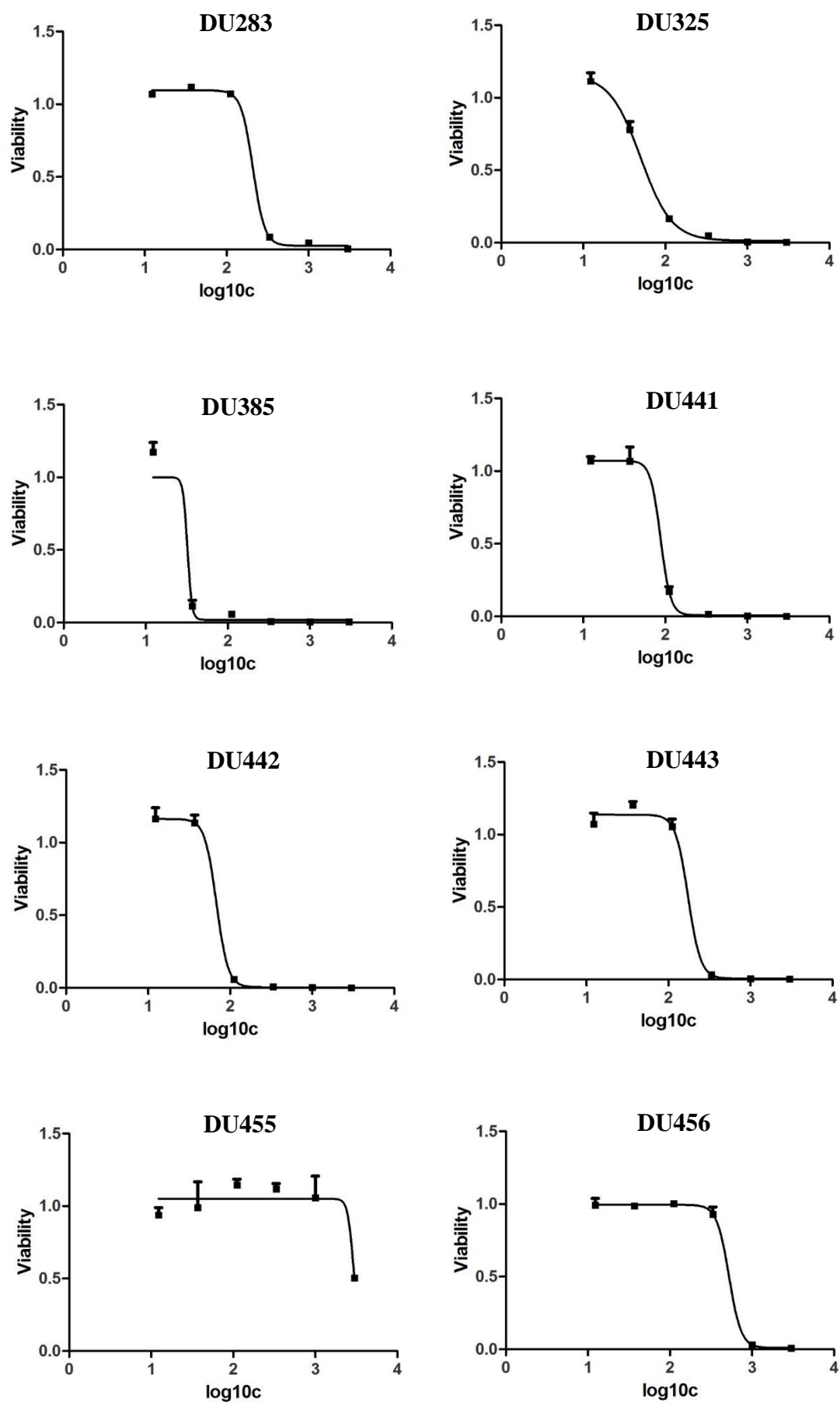

Supplement: Supplementary file 1 [file molecules-23-02845-s001.zip › molecules-372705-SI/Supplement Revised/FigureS4.pdf]

**Figure S5. Dose-response curves of imidazo[1,2-*b*]pyrazole-7-carboxamides on THP-1 cells**

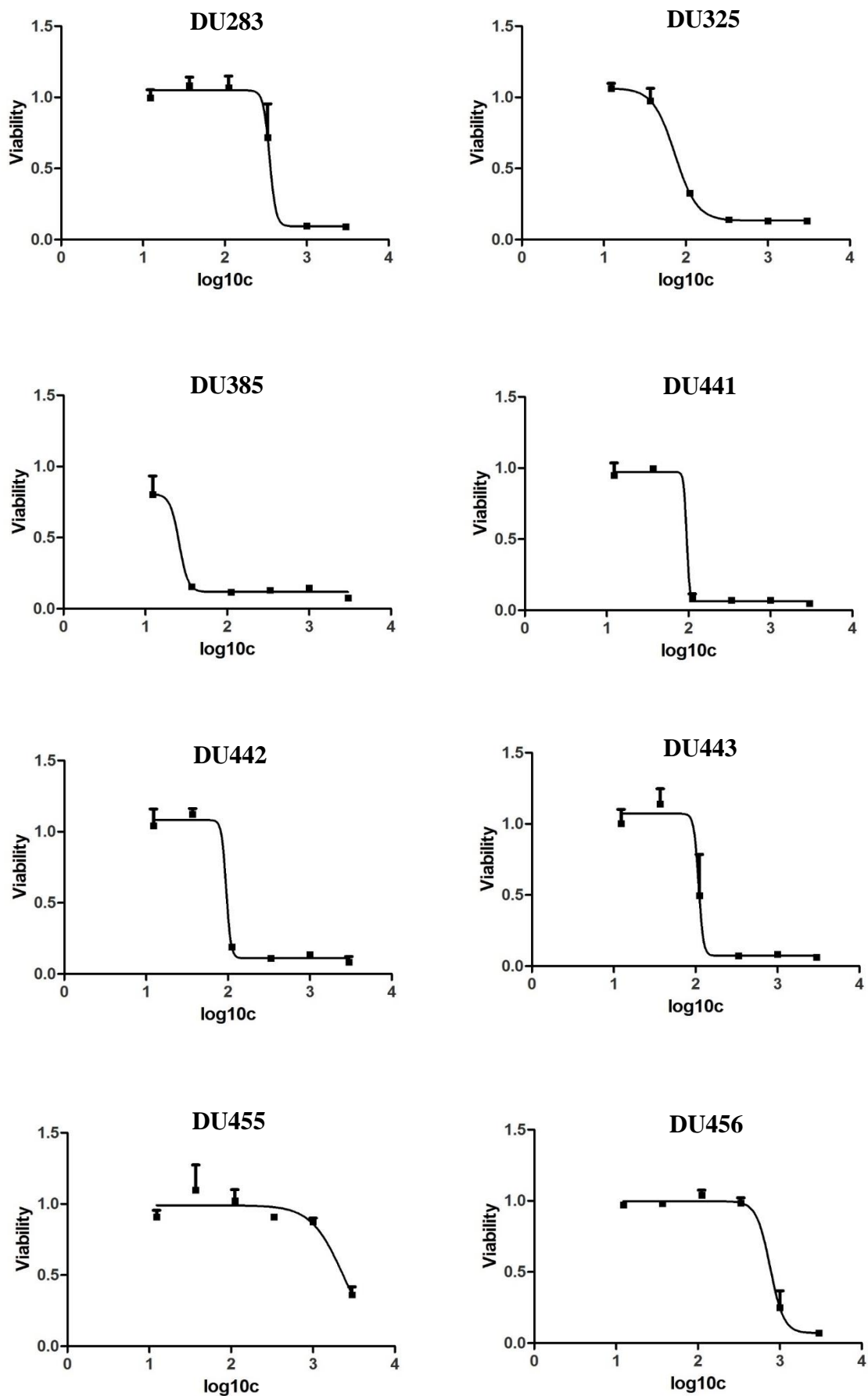

Supplement: Supplementary file 1 [file molecules-23-02845-s001.zip › molecules-372705-SI/Supplement Revised/FigureS5.pdf]

**Figure S6. Dose-response curves of imidazo[1,2-*b*]pyrazole-7-carboxamides on K-562 cells**

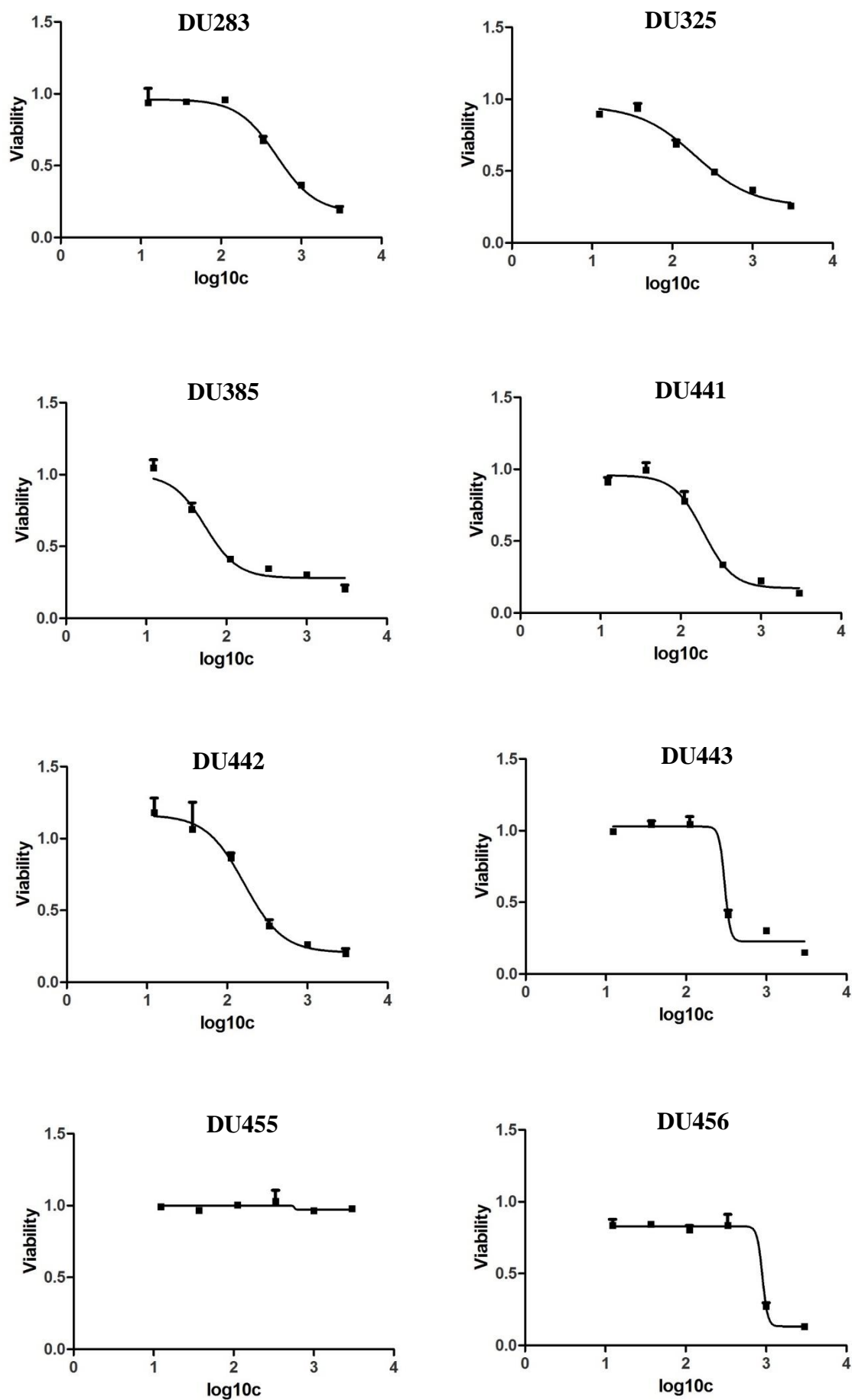

Supplement: Supplementary file 1 [file molecules-23-02845-s001.zip › molecules-372705-SI/Supplement Revised/FigureS6.pdf]

Figure S9. Detection of phosphatidylserine exposure on MV-4-11 cells

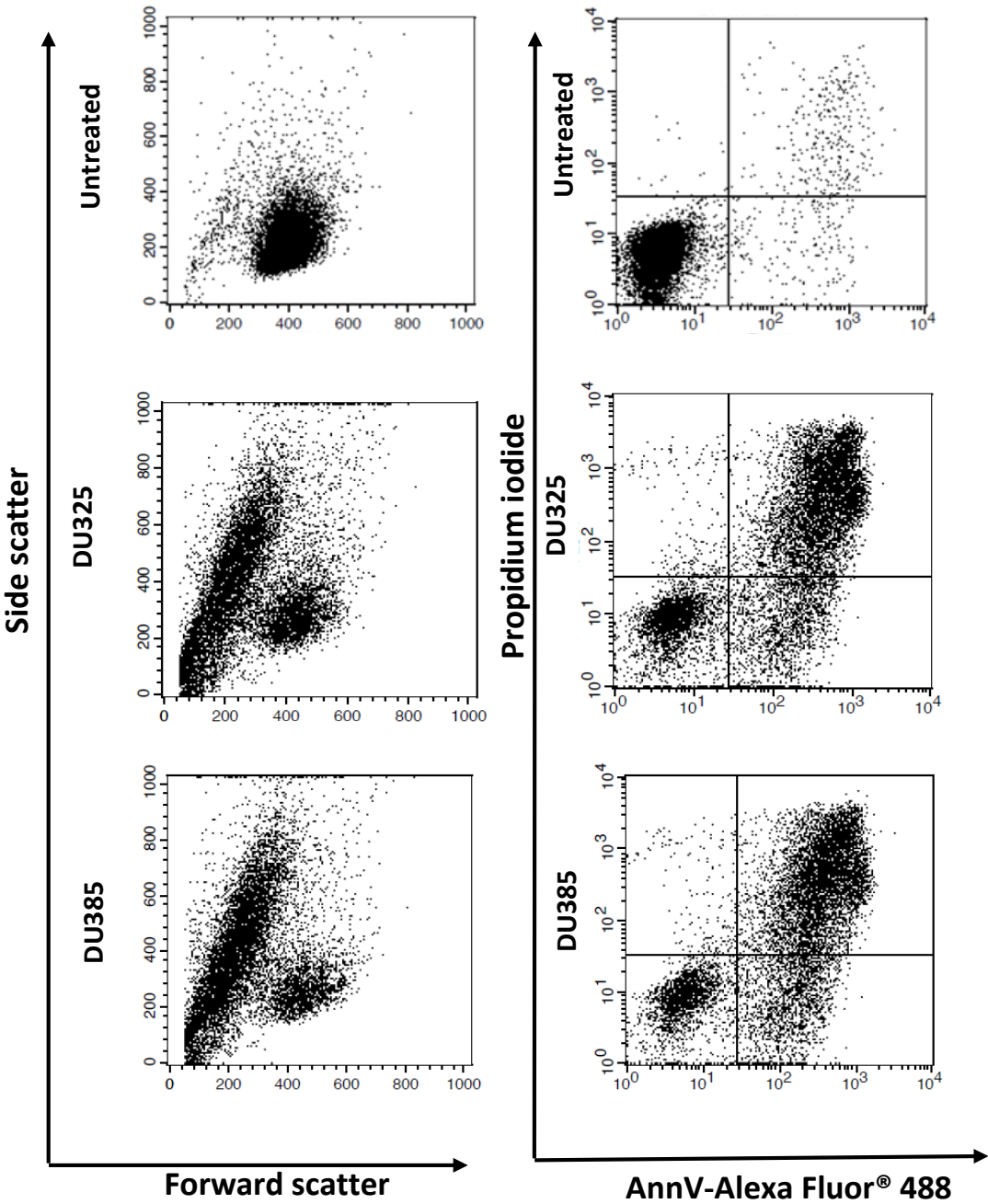

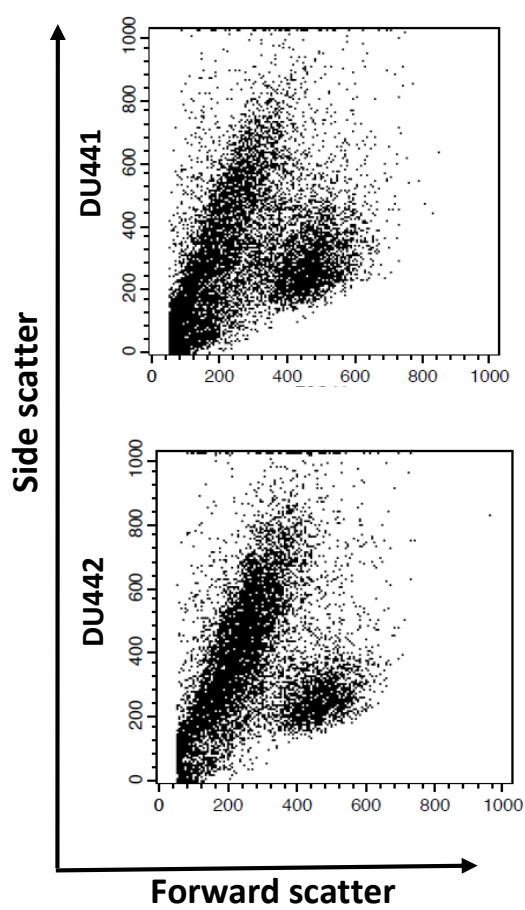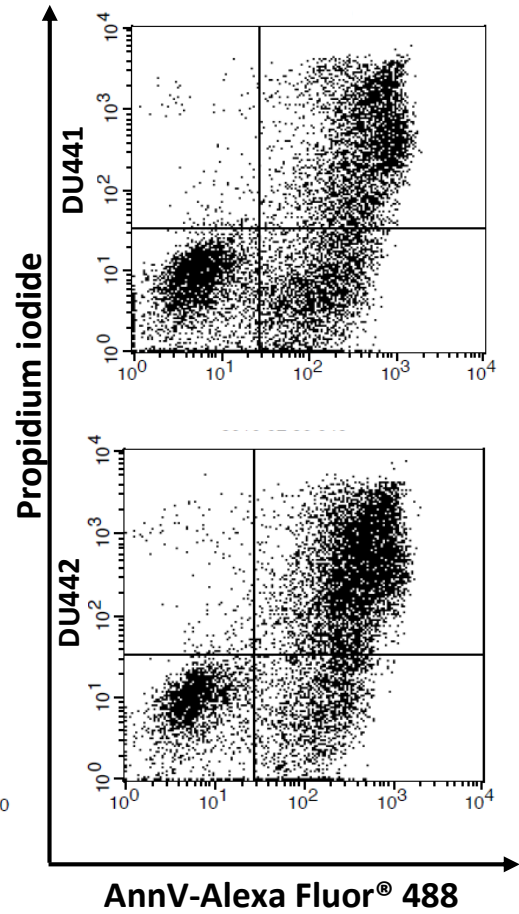

Supplement: Supplementary file 1 [file molecules-23-02845-s001.zip › molecules-372705-SI/Supplement Revised/FigureS9.pdf]
